# Pegylated NIR Fluorophore-Conjugated OBHSA Prodrug for ERα-Targeted Theranostics with Enhanced Imaging and Long-Term Retention

**DOI:** 10.3390/molecules30020305

**Published:** 2025-01-14

**Authors:** Xiaohua Wang, Xiaofei Deng, Lilan Xin, Chune Dong, Guoyuan Hu, Hai-Bing Zhou

**Affiliations:** 1 School of Environmental Ecology and Biological Engineering, Wuhan Institute of Technology, Wuhan 430205, China; msnhm123@gmail.com; 2 College of Life Sciences, Wuchang University of Technology, Wuhan 430223, China; 3 Department of Hematology, Zhongnan Hospital of Wuhan University, Wuhan University School of Pharmaceutical Sciences, Wuhan 430071, China; dxfvca@whu.edu.cn (X.D.); 18537688335@163.com (L.X.); cdong@whu.edu.cn (C.D.); 4 State Key Laboratory of Virology and Biosafety, Frontier Science Center for Immunology and Metabolism, Wuhan University School of Pharmaceutical Sciences, Wuhan 430071, China

**Keywords:** estrogen receptors, drug delivery, NIR fluorescence imaging, prodrug conjugates, pegylation, esterase activation

## Abstract

In recent years, the near-infrared (NIR) fluorescence theranostic system has garnered increasing attention for its advantages in the simultaneous diagnosis- and imaging-guided delivery of therapeutic drugs. However, challenges such as strong background fluorescence signals and rapid metabolism have hindered the achievement of sufficient contrast between tumors and surrounding tissues, limiting the system’s applicability. This study aims to integrate the pegylation strategy with a tumor microenvironment-responsive approach. A novel esterase-activated EPR strategy prodrug, OBHSA-PEG-DCM, was designed. This prodrug links OBHSA, a protein degrader capable of efficient ERα protein degradation, to the PEG-modified fluorescent group (dicyanomethylene-4*H*-pyran, DCM) via an ester bond. This integration facilitates targeted drug delivery and enhances the retention of the fluorescent group within the tumor, allowing distinct in vivo tumor imaging periods. Experimental results show that, benefiting from overexpressed esterase in cancer cells, OBHSA-PEG-DCM can be efficiently hydrolyzed, releasing OBHSA and pegylated DCM. OBHSA demonstrated potent inhibition against MCF-7 cells (IC_50_ = 1.09 μM). Simultaneously, pegylated DCM exhibited remarkable in vivo imaging capabilities, lasting up to 12 days in mice, due to the enhanced permeability and retention (EPR) effect. OBHSA-PEG-DCM holds promise as a theranostic agent for ERα-positive breast cancer, offering both therapeutic and diagnostic capabilities. Importantly, this study highlights the utility of pegylated NIR fluorophores for long-circulating drug delivery systems, addressing current challenges in achieving high-contrast tumor imaging and effective targeted drug release.

## 1. Introduction

Estrogen receptors (ER), encompassing ERα and ERβ, are pivotal in orchestrating target gene expression through signal transduction and transcription factors [1]. ERα, particularly overexpressed in ER-positive (ER^+^) breast cancer (BC), stands as a crucial driver in the formation and progression of ER^+^ BC, making it a prime therapeutic target [2]. Endocrine therapy, a cornerstone in ER^+^ BC treatment, operates by inhibiting estrogen biosynthesis and its interaction with ER [3,4]. However, challenges persist, notably the development of resistance in aromatase inhibitors (AIs) and selective estrogen receptor modulators (SERMs) like letrozole, tamoxifen, and raloxifene [5,6]. Resistance, often fueled by ESR1 gene mutations or crosstalk with other signaling pathways, hampers the clinical utility of SERMs and AIs [7]. Emerging options, such as Selective Estrogen Receptor Degraders (SERDs) like Fulvestrant or Proteolysis Targeting Chimeras (PROTACs), provide primary therapeutic avenues for postmenopausal women facing endocrine-resistant BC or advanced metastatic ERα^+^ BC [8,9,10,11].

Despite this significant progress, real-time monitoring and visualization of ERα protein degradation processes remain challenges in drug development. Integrating biomedical imaging into early drug discovery enhances our understanding of drug mechanisms in both preclinical and clinical research, thereby expediting development [12,13]. In response to this challenge, a near-infrared-based theranostic system has garnered attention [14,15,16,17,18]. The Near-Infrared (NIR) fluorescence theranostic system, a specialized Drug Delivery System (DDS), combines in vivo optical reporters and tumor-specific therapeutic capabilities within a single molecular system. This DDS generates NIR fluorescence signals, enabling real-time monitoring of drug metabolism and distribution and offering insights into local drug dosage and tissue distribution. Noteworthy advantages of this DDS include minimal photodamage to biological samples, deep tissue penetration, and low interference from autofluorescence [19,20]. This technological advancement has made a significant contribution to biomedical research, particularly in elucidating fundamental biological processes. Moreover, to enhance targeting specificity for tumor tissues, bio-conjugated NIR fluorophores have been synthesized. Achieving this specificity involves the incorporation of tumor-directing ligands, such as antibodies, folates, biotin, bevacizumab, and RGD (Arg-Gly-Asp) peptides, into these conjugates [21,22,23].

Despite promising results in multiple trials, achieving tumor images with sufficient contrast between the tumor and surrounding tissues, as well as precise targeted drug release, remains a significant challenge. Many fluorophores utilized in the system exhibit short in vivo half-lives, contributing to nonspecific background signals [24]. Passive accumulation in normal tissues further amplifies nonspecific background signals. Additionally, most labeled fluorophores may not reach specific tumor sites, leading to prolonged background fluorescence. The time required to clear this background interference is often overlooked, introducing uncertainty in practical applications and raising questions about the overall benefits of actively targeting tumors with fluorescence probes. These challenges motivate us to explore the potential of pegylated NIR fluorophores, as they may offer very long biological half-lives and an economical passive cancer-targeting delivery system. The considerable duration of these biological half-lives holds the promise of a prolonged waiting period to eliminate background fluorescence interference, thereby facilitating the acquisition of tumor images with sufficient contrast between cancerous and normal tissues. The covalent attachment of polyethylene glycols (PEG) to a drug molecule or delivery vehicle, initially reported in the late 1970s, has been widely used in the pharmaceutical industry to enhance the clinical performance of various drug candidates [25,26]. Pegylation designates the covalent attachment of one (or more) PEG chains, either to a low-molecular-weight drug, a large biomolecule, or to the delivery vector for the drug molecule, such as liposomes or nanoparticles [27]. Furthermore, the use of pegylated fluorophores is advantageous, as PEG groups are recognized for their ability to enhance fluorescence quantum yields in aqueous environments [24]. Furthermore, exploiting the unique tumor microenvironment (TME) to design prodrugs represents a promising approach for cancer therapeutics. By leveraging the disparities between the TME and the normal cell microenvironment, these prodrugs can achieve enhanced tumor targeting and selectivity, thereby minimizing systemic toxicity. Common TME factors that can be exploited for prodrug design include the following: elevated intracellular thiols, lower pH than normal cells, a reactive oxygen species (ROS) environment, hypoxia, and overexpression of specific enzymes, etc. [28,29]. Esterase is overexpressed in cancer cells and plays a critical role in tumor invasion, migration, and growth, making it a promising target for cancer therapy [30,31].

**Scheme 1:**
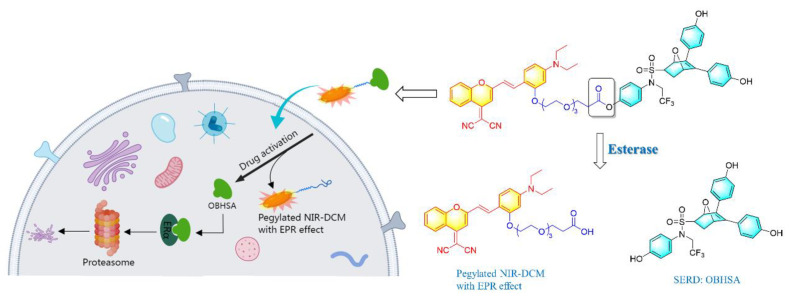
Chemical structure of prodrug OBHSA-PEG-DCM and its working mechanism in the presence of esterase.

Given the combined concerns above, we designed an esterase-activated prodrug, OBHSA-functionalized pegylated near-infrared DCM (OBHSA-PEG-DCM), aiming for the precise targeted degradation of ERα and achieving sufficient contrast for image-guided cancer therapy, in which 7-oxabicyclo [2.2.1]heptene sulfonamide (OBHSA), a novel ER degrader pharmacophore skeleton possessing a unique mechanism developed by us previously, was selected as the ER-targeting and degradation warhead [32], while dicyanomethylene-4*H*-pyran (DCM) chromophores were chosen for their excellent photophysical properties, characterized by heightened sensitivity to electron disturbance, emissions of longer wavelengths (red light), and a high fluorescent yield, along with high photostability [33]. OBHSA-PEG-DCM was synthesized by conjugating the ERα degrader OBHSA with fluorophore DCM through a hydrolyzable ester bond (Scheme 1). Experimental results demonstrated that, due to the overexpression of esterase in cancer cells, the ester group of OBHSA-PEG-DCM underwent selective hydrolysis, leading to the release of OBHSA and pegylated DCM, thus exhibiting potent inhibition against MCF-7 cells (IC_50_ = 1.09 ± 0.08 μM) and distinct ERα degradation activity. Simultaneously, pegylated DCM exhibited remarkable in vivo imaging capabilities in mice, due to the enhanced permeability and retention (EPR) effect. It maintained a sustained high tumor-to-background ratio, reaching peak tumor emission within 2 h and lasting for up to 2 weeks in mice bearing MCF-7 xenografts. In summary, OBHSA-PEG-DCM is a prodrug capable of efficiently targeting and degrading ERα protein, while also possessing high contrast and excellent imaging capability for visualization-based therapy. Furthermore, the significance of this study lies in it providing a novel approach for utilizing PEG-modified near-infrared fluorescent probes as a strategy for long-circulating drug delivery systems.

## 2. Results and Discussion

### 2.1. Synthetic Methodology

**Scheme 2:**
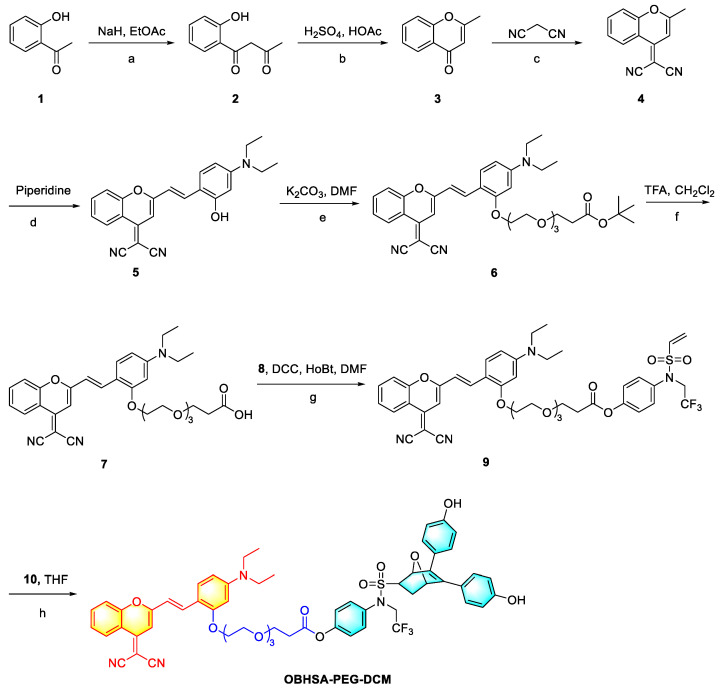
Synthetic route for OBHSA-PEG-DCM. Reagents and conditions: (**a**) NaH, EtOAc, THF, and reflux; (**b**) HOAc, H_2_SO_4_, and reflux; (**c**) malononitrile, Ac_2_O, and reflux; (**d**) 4-(Diethylamino)-2-hydroxybenzaldehyde, toluene, piperidine, HOAc, and reflux; (**e**) *tert*-butyl 3-(2-(2-(2-bromoethoxy)-ethoxy)ethoxy)propanoate, K_2_CO_3_, and DMF; (**f**) TFA, CH_2_Cl_2_, and rt; and (**g**) DCC, HOBt, DIPEA, DMF, rt; (**h**) 4,4′-(Furan-3,4-diyl)diphenol, THF, and reflux.

The synthesis of OBHSA-PEG-DCM is depicted in Scheme 2. Critical intermediates, including dicyanomethylene-*4H*-pyran (DCM) derivative **5**, ethylene sulfonamide derivative **8,** and the furan intermediate **10**, were synthesized by adopting well-established protocols with appropriate modifications [34,35,36]. For experimental details, see pages S3–S4 of the Supplementary Materials. To synthesize OBHSA-PEG-DCM, compound **5** was initially coupled with *tert*-butyl 3-(2-(2-(2-bromoethoxy)ethoxy)-ethoxy)propanoate through Williamson ether synthesis, resulting in the formation of intermediate **6**. Subsequently, intermediate **6** underwent deprotection in the presence of trifluoroacetic acid, leading to the generation of carboxylic acid **7**. Carboxylic acid **7** was then condensed with ethylene sulfonamide derivative **8** in the presence of DCC and HOBt, yielding compound **9**. The final product, OBHSA-PEG-DCM, was obtained through a Diels–Alder reaction between compounds **9** and **10**. All synthesized compounds were characterized using NMR and high-resolution mass spectrometry.

**Figure 1:**
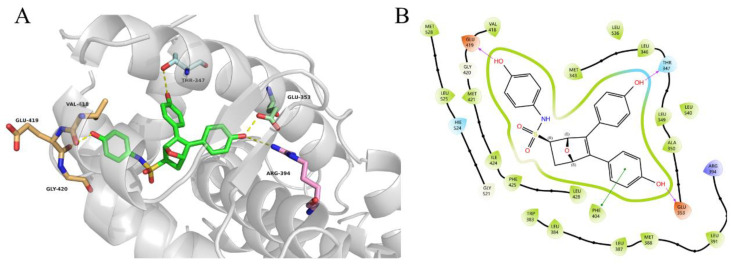
Molecular docking results of the (1*S*,4*S*) enantiomer of OBHSA with ERα (PDB: 5KCC). (**A**) Analysis of the docking interactions between the (1*S*,4*S*) enantiomer of OBHSA and Erα. (**B**) Schematic representation of the interaction between the residues of the ERα ligand-binding domain and the (1*S*,4*S*) enantiomer of OBHSA.

It is worth noting that, as we have previously observed, the Diels–Alder reaction predominantly yielded thermodynamically favorable *exo* products [37,38,39]. In this study, the *exo* product was more easily generated through this Diels–Alder reaction, likely due to the high rate and easy reversibility of the reaction, while the *endo* isomers were hardly found. The ratios of regioisomers were determined from the corresponding ^1^H NMR spectra, and the structures and stereoisomeric assignments of the single isomers were analyzed by NOESY-NMR (see Figure S2 of Supplementary Materials). Additionally, we conducted molecular docking studies on the four possible stereoisomers. As shown in Figure 1, the docking results indicate that only the (1*S*,4*S*) enantiomer can favorably interact with the ERα protein (PDB: 5KCC [40]).

### 2.2. Photophysical Properties

**Figure 2:**
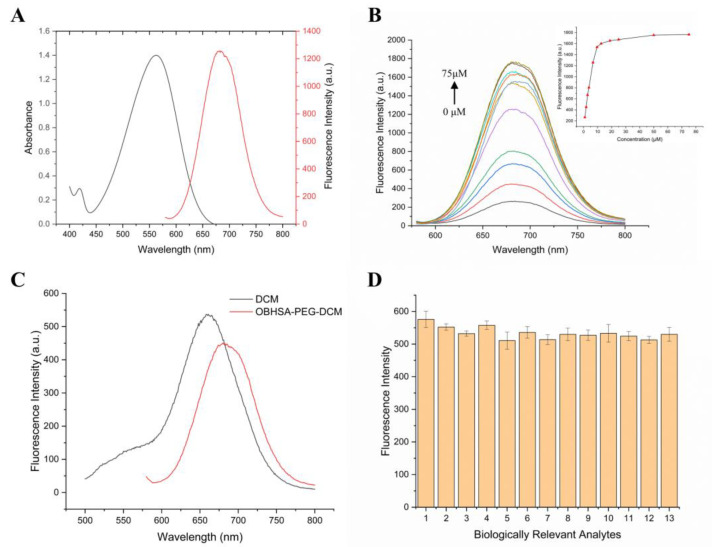
Photophysical properties of the system. (**A**) Absorption profiles at a concentration of 50 μM and emission profiles at a concentration of 10 μM in DMSO/PBS (10/90, *v*/*v*, pH = 7.4). (**B**) Emission changes at a concentration of 10 μM in DMSO/PBS (10/90, *v*/*v*, pH = 7.4). Inset (**B**) Relationship between fluorescence intensity at 682 nm and concentration. (**C**) Fluorescence spectra of DCM (10 µM) and OBHSA-PEG-DCM (10 µM with 0.2 U/mL of esterase) in DMSO/PBS solution (1:1, *v*/*v*, pH = 7.4, 10 mM). (**D**) Fluorescence responses to various cellular analytes. 1. blank; 2. NaCl; 3. KCl; 4. MgCl_2_; 5. CaCl_2_; 6. NaNO_2_; 7. NaHCO_3_; 8. Cys; 9. Ser; 10. Leu; 11. HClO; 12. ^1^O_2_; 13. ⋅OH.

To assess the anticipated OBHSA-PEG-DCM imaging capabilities of the system, we analyzed its photophysical properties. The absorption spectrum showed that the probe exhibited its primary absorption peak at 562 nm. Simultaneously, upon excitation at 562 nm, it exhibited a maximum emission wavelength (λem) of 682 nm, with a large Stokes shift of 120 nm (Figure 2A). As shown in Figure 2B, within the concentration range of 0–75 μM, the fluorescence intensity increases with rising concentration. However, when the concentration exceeds 75 μM, fluorescence quenching or saturation occurs, resulting in a lack of further increase in fluorescence intensity with increasing concentration. Subsequently, we compared the spectra of free DCM with those of OBHSA-PEG-DCM after treatment with esterase. While the positions and shapes of the spectra exhibited a high degree of similarity, they were not identical. This discrepancy may be attributed to the influence of the polyethylene glycol chain on the optical properties of the fluorescent group DCM. Furthermore, their fluorescence intensities were found to be comparable (Figure 2C).

To evaluate the potential application of OBHSA-PEG-DCM in real biological systems, we investigated its reactions with various biologically relevant analytes, including reactive species such as HClO, ^1^O_2_, and ⋅OH, along with amino acids and abundant metal ions. This involved 100 μM cations, including Na^+^, K^+^, Mg^2+^, and Ca^2+^, and 100 μM anions, such as Cl^−^, NO_2_^−^, and HCO_3_^−^. Furthermore, this selectivity extended to amino acids like Cys, Ser, and Leu under similar conditions, aiming to assess possible interferences. As depicted in Figure 2D, OBHSA-PEG-DCM exhibited minimal changes in fluorescence under these conditions.

### 2.3. Hydrolyzed Product and Mechanism Investigation

**Figure 3:**
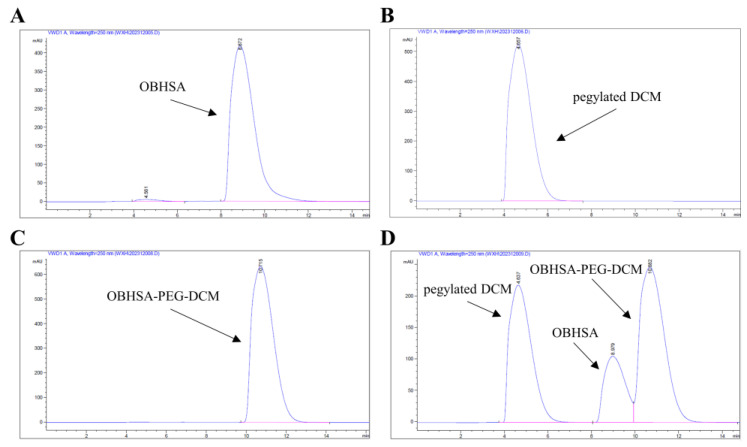
HPLC analysis of cell lysates after co-incubation of 10 µM OBHSA-PEG-DCM with MCF-7 cells for 45 min. (**A**) OBHSA. (**B**) Pegylated DCM. (**C**) OBHSA-PEG-DCM. (**D**) Cellular lysate.

In this study, we also investigated the intracellular release mechanism of OBHSA-PEG-DCM within tumor cells, with a specific focus on the release of pegylated DCM fluorescent groups and OBHSA. To achieve this, we extracted cellular lysates and performed both HPLC and HRMS analyses. Our results revealed that the retention time of OBHSA-PEG-DCM was found to be 10.715 min, as depicted in Figure 3. Over time, the peak area corresponding to the 10.715 min decreased in the cellular lysates extracted 45 min later. Simultaneously, two new peaks emerged at retention times of 4.657 min and 8.872 min, corresponding to pegylated DCM and OBHSA, respectively. Notably, after 12 h of monitoring the cellular lysates, the original 10.715 min peak nearly vanished. Collectively, these findings demonstrate that OBHSA-PEG-DCM undergoes cleavage, releasing both OBHSA and PEG-modified DCM.

Further HRMS analysis revealed two new molecular ion peaks with *m*/*z* values of 534.1190 and 588.2711, corresponding to OBHSA and PEG-modified DCM, respectively (Supplementary Materials Figure S1). Both HPLC and HRMS experimental results affirm the successful release of OBHSA and PEG-modified DCM catalyzed by overexpressed esterase.

### 2.4. Evaluation of Inhibitory and Degradation Activities

**Figure 4:**
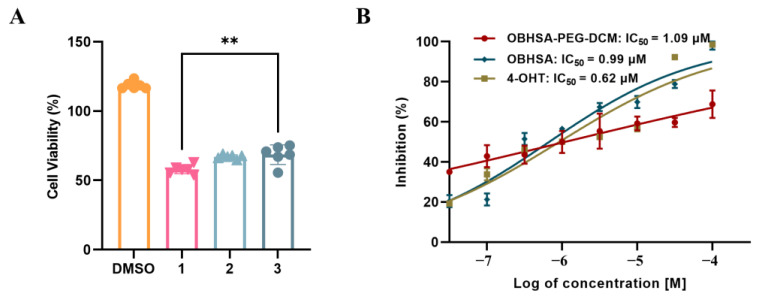
Cell viability curve. (**A**) Cell viability assay on MCF-7 cells. 1. OBHSA (1 μM); 2. OBHSA-PEG-DCM (1 μM) + esterase (0.1 U/mL); and 3. OBHSA-PEG-DCM (1 μM). (**B**) IC_50_ of OBHSA-PEG-DCM and 4-OHT on MCF-7 cells. Experimental values represent an average of at least three independent experiments ± standard deviation (mean ± SD), ** *p* < 0.01.

Given that OBHSA-PEG-DCM can be activated to release OBHSA, we investigated its performance in MCF-7 cells in vitro. The proliferation inhibition of free OBHSA and OBHSA-PEG-DCM was evaluated and the results are summarized in Figure 4 using the CCK-8 (Cell Counting Kit 8) assay. Compared with the parent drug OBHSA, OBHSA-PEG-DCM showed similar cytotoxicity, with significant in vitro antiproliferative activity against MCF-7 cells (Figure 4A). The overexpression of esterase in MCF-7 cells may contribute to this result. Compared to OBHSA, OBHSA-PEG-DCM exhibited similar cytotoxicity in MCF-7 cells, with an IC_50_ value of 1.09 ± 0.08 μM, slightly higher than that of 4-OHT (0.62 μM) (Figure 4B). Based on our previous research [41], we found that the IC_50_ value of OBHSA for MCF-7 cells was 10.5 μM. After modifying OBHSA with a PEG chain to form OBHSA-PEG-DCM, the IC_50_ value decreased to 1.09 μM. This reduction may be attributed to the longer PEG chain, which extends the drug’s retention time within the cells.

To better validate our findings and demonstrate the specificity of our prodrug, we included a broader range of human tumor cell lines. The study assessed the cytotoxicity of free OBHSA and OBHSA-PEG-DCM against a larger panel of human tumor cell lines, including ERα-negative breast cancer MDA-MB-231 and prostate cancer DU145. The IC_50_ values of OBHSA-PEG-DCM were similar to the parent drug OBHSA in MCF-7 cells and were significantly lower in MDA-MB-231. However, both compounds showed much lower cytotoxicity in DU145 cells, with IC_50_ values greater than 100 µM (Table 1).

**Figure 5:**
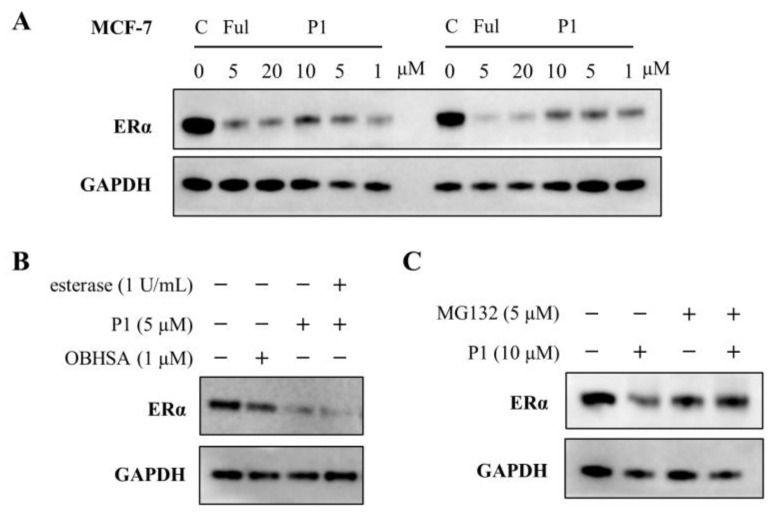
Investigation of the degradation activity of OBHSA-PEG-DCM (hereafter referred to as **P1**). (**A**) Evaluation of the degradation activity of **P1** by Western blot assay. (**B**) Evaluation of the degradation activity of **P1** and OBHSA. (**C**) Investigation of the degradation mechanism of **P1**. MCF-7 cells were treated with compound **P1**, MG132, or their combination for 24 h, and the ERα level was then analyzed.

Consistent with the inhibitory activity, OBHSA-PEG-DCM also demonstrated high degradation activity in MCF-7 cells (Figure 5A). The parent compound OBHSA is a novel selective estrogen receptor degrader (SERD) that can effectively inhibit MCF-7 cell proliferation and exhibits significant ERα degradation efficacy [32]. To evaluate the degradation activity of OBHSA-PEG-DCM, we also compared the degradation activities of OBHSA and OBHSA-PEG-DCM after esterase-accelerated treatment. Upon the addition of 0.1 U/mL esterase for acceleration [30], the results showed that the degradation activity of OBHSA-PEG-DCM was improved and was basically consistent with the degradation activity of OBHSA (Figure 5B). This further indicates that excessive esterase can induce the hydrolysis of OBHSA-PEG-DCM. In addition, we also found that the proteasome inhibitor MG132 could partially block the ERα degradation induced by OBHSA-PEG-DCM, while MG132 alone had no effect on the expression of ERα protein. These results indicate that the degradation mechanism of OBHSA-PEG-DCM is derived from the proteasome-dependent mechanism of OBHSA (Figure 5C).

### 2.5. Cellular and Animal Imaging Investigations

**Figure 6:**
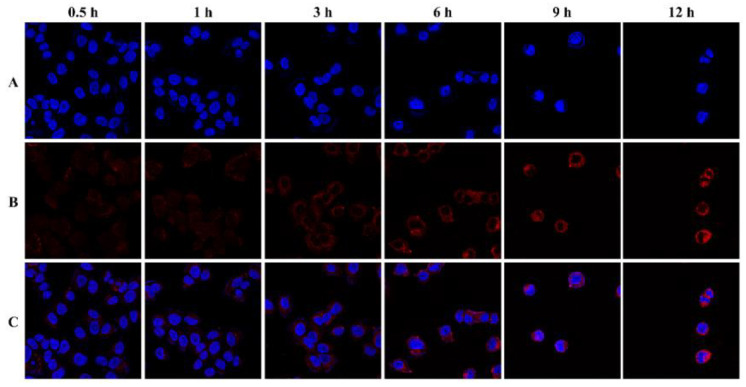
CLSM imaging analysis of OBHSA-PEG-DCM at 1.0 μM over various time points. (**A**) DAPI was used for intracellular localization (blue). (**B**) Fluorescence image of MCF-7 cells incubated with OBHSA-PEG-DCM (10.0 μM). (**C**) Overlay of the merged images from (**A**,**B**). Cellular images were captured using excitation wavelengths at 556 nm and a band-pass emission filter of 650–750 nm. Scale bar: 50 μm.

Building upon the encouraging results above, we further investigated the in vitro and in vivo imaging capabilities of OBHSA-PEG-DCM in MCF-7 cells and mouse models, respectively. Initially, confocal laser scanning microscopy (CLSM) was employed to assess the cellular uptake and distribution of OBHSA-PEG-DCM. MCF-7 cells were co-cultured with 1.0 μM OBHSA-PEG-DCM, and the corresponding fluorescence images were acquired. As illustrated in Figure 6, red fluorescence was observed in the cytoplasm after 30 min of incubation at 37 °C, indicating the efficient cellular uptake of OBHSA-PEG-DCM by MCF-7 cells. Over time, the fluorescence intensity gradually increased, demonstrating the enhanced cellular uptake of OBHSA-PEG-DCM. Concomitantly, a decrease in the number of MCF-7 cells was observed, consistent with the ERα degradation effect of OBHSA-PEG-DCM.

**Figure 7:**
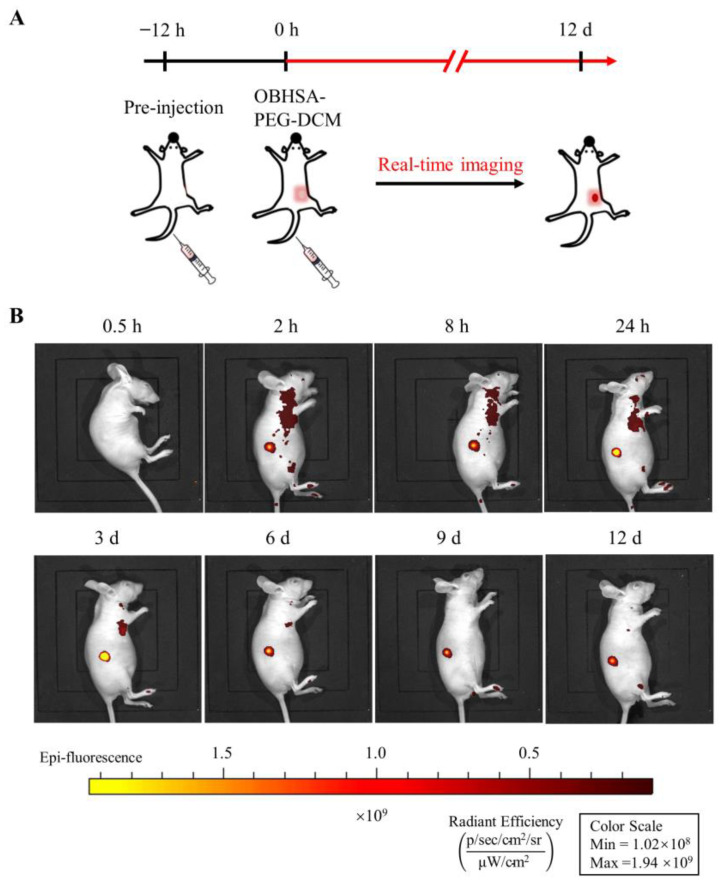
In vivo real-time NIR imaging of MCF-7 tumor-bearing BALB/c mice. (**A**) Timeline of OBHSA-PEG-DCM injection and NIR imaging. (**B**) NIR imaging was performed at various timepoints post injection of OBHSA-PEG-DCM (0.05 mg/kg).

Subsequently, in vivo imaging experiments were conducted to explore the imaging potential of OBHSA-PEG-DCM in living systems. OBHSA-PEG-DCM was administered via tail vein injection to six-week-old BALB/c mice bearing MCF-7 breast cancer xenografts. As shown in Figure 7, no fluorescence signal was detected within 30 min after OBHSA-PEG-DCM injection. However, fluorescence emerged at the tumor site 2 h post injection, and the fluorescence intensity increased over time. Notably, the strongest fluorescence signal was observed throughout the entire tumor region at 24 h. Importantly, no fluorescence signal was detected from the other organs of the mice, indicating the excellent tumor specificity of OBHSA-PEG-DCM. To further validate the long-term retention of OBHSA-PEG-DCM at tumor sites, the imaging time of the mice was extended to 2 weeks. The results demonstrated that a relatively strong fluorescence signal could still be detected at the tumor site after two weeks. In our current study, the primary focus is exploring the feasibility of integrating a pegylation strategy with a tumor microenvironment-responsive approach for prodrug design. As such, we did not include OBHSA as a direct control for the in vivo therapeutic efficacy. Collectively, these findings suggest that OBHSA-PEG-DCM possesses excellent tumor specificity and prolonged retention capability, highlighting its potential as a theranostic agent for real-time fluorescence-guided diagnosis and treatment.

### 2.6. H&E Staining

**Figure 8:**
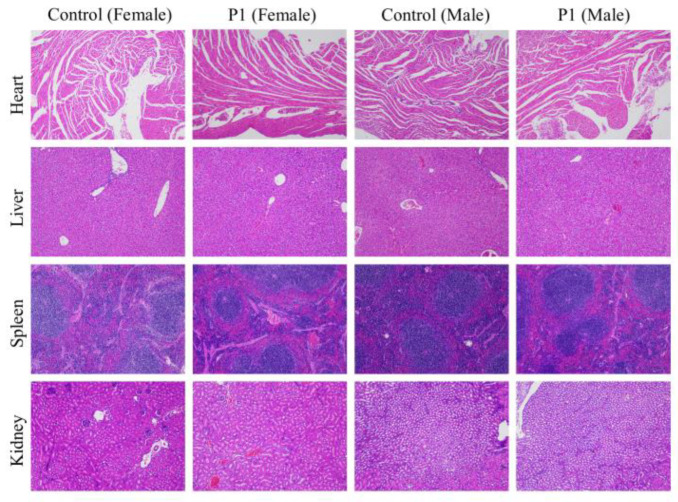
H&E staining of major organs after treatment. The experimental group received the drug via intraperitoneal injection at a dosage of 100 mg/kg. The negative control group received an intraperitoneal injection of a solution containing 5% DMSO, 45% PEG, and 50% PBS. Scale bar: 100 µm.

The histological analysis of tissue sections from various major organs isolated from mice post sacrifice was conducted using H&E staining, to determine the extent of tissue damage in these organs. As shown in Figure 8, no tissue damage or toxicity was observed in any of the major organs compared to the control group.

## 3. Conclusions

This study successfully designed and evaluated a novel ERα-targeted theranostic prodrug, OBHSA-PEG-DCM. This innovative prodrug integrates the advantages of ERα degradation with a pegylated near-infrared (NIR) fluorophore, enabling real-time monitoring of the therapeutic process. Key findings demonstrate that due to the overexpression of esterase in cancer cells, OBHSA-PEG-DCM undergoes efficient hydrolysis, leading to the release of OBHSA and the pegylated NIR fluorophore (pegylated DCM). OBHSA-PEG-DCM effectively degrades ERα protein in MCF-7 breast cancer cells, exhibiting activity similar to the parent drug OBHSA. In vitro studies confirm the efficient cellular uptake and prolonged retention of OBHSA-PEG-DCM by MCF-7 cells. Furthermore, in vivo imaging studies reveal the excellent tumor targeting and specificity of OBHSA-PEG-DCM in mice bearing MCF-7 xenografts. The pegylated NIR fluorophore facilitates a sustained and high tumor-to-background ratio, allowing for the clear visualization of tumors over an extended period. Significantly, the pegylation strategy enhances the circulation time of OBHSA-PEG-DCM, highlighting its potential as a long-circulating drug delivery system.

These findings collectively suggest that OBHSA-PEG-DCM holds great promise as a theranostic agent for ERα-positive breast cancer. It offers the combined benefits of targeted therapy, real-time monitoring through fluorescence imaging, and a long-circulating drug delivery system. This work paves the way for further exploration of pegylated NIR fluorophores as a strategy for designing long-circulating drug delivery systems for cancer theranostics. Future studies could focus on optimizing the linker between the therapeutic moiety and the fluorophore for controlled drug release, potentially leading to further enhanced therapeutic efficacy.

## 4. Experimental Section

### 4.1. Synthesis of OBHSA-PEG-DCM

Chemical reagents were commercially available and of analytical purity. Reaction progress was monitored using thin-layer chromatography, and purification was carried out using 200–400 mesh silica gel columns. NMR spectra (^1^H and ^13^C) were recorded using Bruker Biospin AV400 (400 MHz) NMR spectrometers (Bruker Biospin, Billerica, MA, USA), with TMS as an internal standard and DMSO-*d*_6_ as the solvent. Mass spectra were obtained through an Ionspec 4.7 T FTMS mass spectrometer (Ionspec, Orlando, FL, USA). Key intermediates, including compounds **5**, **8**, and **10**, were synthesized using established protocols with necessary modifications. Experimental details can be found on pages S3–S4 in Supplementary Materials.

*Tert*-Butyl (*E*)-3-(2-(2-(2-(2-(2-(4-(dicyanomethylene)-4*H*-chromen-2-yl)vinyl)-5-(diethylamino)phenoxy)ethoxy)ethoxy)ethoxy)propanoate (**6**):

Compound **5** (350 mg, 912.8 mmol) and *tert*-butyl 3-(2-(2-(2-bromoethoxy)-ethoxy)ethoxy)propanoate (342.6 mg, 1.0 mol) were dissolved in DMF (50 mL) with K_2_CO_3_ (252.3 mg, 1.83 mol) and stirred for 4–5 h at 50 °C. The reaction mixture was poured into water (150 mL) and extracted with CH_2_Cl_2_ (3 × 50 mL). The combined organic layer was washed with water and brine, dried over anhydrous Na_2_SO_4_, filtered, and concentrated. The resulting crude product was purified by column chromatography (CH_2_Cl_2_/MeOH = 50:1) to yield **6** (524.74 mg, yield: 89%). ^1^H NMR (400 MHz, DMSO-*d*_6_) δ 8.74–8.67 (m, 1H), 7.92–7.84 (m, 2H), 7.72–7.65 (m, 1H), 7.59–7.52 (m, 2H), 7.09 (d, *J* = 15.8 Hz, 1H), 6.74 (s, 1H), 6.40–6.35 (m, 1H), 6.21 (d, *J* = 2.3 Hz, 1H), 4.25–4.18 (t, *J* = 4.5 Hz, 2H), 3.91–3.84 (t, *J* = 4.4 Hz, 2H), 3.74–3.67 (m, 2H), 3.62–3.55 (m, 2H), 3.52–3.41 (m, 11H), 2.36–2.28 (t, *J* = 6.2 Hz, 2H), 1.34 (s, 9H), and 1.16–1.09 (t, *J* = 6.9 Hz, 6H). ^13^C NMR (151 MHz, DMSO-*d*_6_) δ 170.3, 162.3, 160.4, 159.8, 152.2, 152.0, 151.3, 135.8, 134.8, 131.1, 125.8, 124.5, 118.7, 118.0, 117.3, 116.7, 112.0, 111.2, 105.1, 104.3, 94.7, 79.6, 70.2, 70.0, 69.7, 69.6, 69.0, 67.8, 66.2, 56.3, 54.9, 48.6, 44.0, 35.8, 30.8, 27.7, and 12.6.

(*E*)-3-(2-(2-(2-(2-(2-(4-(Dicyanomethylene)-4*H*-chromen-2-yl)vinyl)-5-(diethylamino)phenoxy)ethoxy)ethoxy)ethoxy)propanoic acid (**7**):

Compound **6** (500 mg) was dissolved in a 100 mL solution of anhydrous CH_2_Cl_2_ in an ice bath. Subsequently, 10 mL of trifluoroacetic acid (TFA) was added dropwise. The reaction mixture was warmed to room temperature and stirred for 2 h. Following this, the mixture was washed with brine, and the organic layer was evaporated under reduced pressure. The resulting crude product was then subjected to purification by column chromatography (CH_2_Cl_2_/MeOH = 50:1), yielding compound **7** (424.47 mg, yield: 93%). ^1^H NMR (400 MHz, DMSO-*d*_6_) δ 8.70 (d, *J* = 8.2 Hz, 1H), 7.91–7.82 (m, 2H), 7.68 (d, *J* = 8.3 Hz, 1H), 7.60–7.52 (m, 2H), 7.08 (d, *J* = 15.8 Hz, 1H), 6.73 (s, 1H), 6.41–6.33 (m, 1H), 6.19 (d, *J* = 2.3 Hz, 1H), 4.25–4.18 (m, 2H), 3.91–3.84 (m, 2H), 3.75–3.68 (m, 2H), 3.62–3.55 (m, 3H), 3.54–3.44 (m, 9H), 2.39–2.32 (t, *J* = 6.4 Hz, 2H), and 1.16–1.10 (t, *J* = 6.9 Hz, 6H).

*N*-(4-Hydroxyphenyl)-*N*-(2,2,2-trifluoroethyl)ethenesulfon-amide (**8**):

Key intermediate **8** was synthesized by adopting well-established protocols with appropriate modifications^33^. ^1^H NMR (400 MHz, DMSO-*d*_6_) δ 9.81 (s, 1H), 7.21–7.14 (m, 2H), 7.01–6.90 (m, 1H), 6.78 (d, *J* = 8.2 Hz, 2H), 6.14 (d, *J* = 9.5 Hz, 1H), 6.01 (d, *J* = 16.0 Hz, 1H), and 4.43–4.32 (t, *J* = 9.3 Hz, 2H).

4-(*N*-(2,2,2-Trifluoroethyl)vinylsulfonamido)phenyl (*E*)-3-(2-(2-(2-(2-(2-(4-(dicyanomethylene)-4*H*-chromen-2-yl)vinyl)-5-(diethylamino)phenoxy)ethoxy)-ethoxy)ethoxy)propanoate (**9**):

Under an N_2_ atmosphere, compound **7** (320 mg, 544.52 μmol) was dissolved in 50 mL anhydrous DMF. Subsequently, DCC (134.82 mg, 653.82 μmol) and HOBt (88.29 mg, 653.82 μmol) were added to the reaction mixture. Then, compound **8** (153.2 mg, 544.52 μmol) and DIPEA (281.51 mg, 2.18 mmol) were added at 0 °C sequentially. The reaction mixture was stirred for 10 min, followed by warming to room temperature and stirring for an additional 12 h. Upon completion of the reaction, the mixture was quenched with water and extracted with CH_2_Cl_2_. The combined organic layers were washed with water and brine, dried over anhydrous Na_2_SO_4_, filtered, and concentrated. The crude product was purified by column chromatography (CH_2_Cl_2_/MeOH = 150:1) to afford compound **9** (194.6 mg, yield: 42%). ^1^H NMR (400 MHz, DMSO-*d*_6_) δ 8.73–8.66 (m, 1H), 7.92–7.81 (m, 2H), 7.71–7.64 (m, 1H), 7.59–7.50 (t, *J* = 7.7 Hz, 2H), 7.43–7.37 (m, 2H), 7.18–7.07 (m, 3H), 7.02–6.92 (m, 1H), 6.75 (s, 1H), 6.38 (m, 1H), 6.20 (d, *J* = 2.3 Hz, 1H), 6.15 (d, *J* = 9.9 Hz, 1H), 6.03 (d, *J* = 16.4 Hz, 1H), 4.51–4.41 (t, *J* = 8.8 Hz, 2H), 4.21 (d, *J* = 5.1 Hz, 2H), 3.91–3.84 (t, *J* = 4.6 Hz, 2H), 3.75–3.69 (m, 2H), 3.68–3.63 (t, *J* = 6.2 Hz, 2H), 3.62–3.58 (m, 2H), 3.55–3.44 (m, 8H), 2.77–2.71 (m, 2H), and 1.16–1.10 (t, *J* = 6.9 Hz, 6H). ^13^C NMR (151 MHz, DMSO-*d*_6_) δ 169.7, 162.3, 160.3, 159.8, 152.2, 152.0, 151.3, 150.1, 136.4, 135.8, 134.8, 133.7, 131.1, 130.2, 129.8, 125.7, 124.5 (q, *J* = 152.7 Hz), 122.6, 118.6, 118.0, 117.3, 116.7, 112.0, 111.2, 105.1, 104.3, 94.7, 70.2, 70.0, 69.7, 69.0, 67.7, 65.8, 56.3, 54.9, 51.4 (q, *J* = 70.6 Hz), 44.0, 35.8, 34.7, 30.8, 16.9, 16.4, and 12.6. ^19^F NMR (376 MHz, DMSO-*d*_6_) δ −69.6.

4,4′-(Furan-3,4-diyl)diphenol (**10**):

Key intermediate **10** was synthesized by adopting well-established protocols with appropriate modifications^35^. ^1^H NMR (400 MHz, DMSO-*d*_6_) δ 9.45 (s, 2H), 7.74 (s, 2H), 7.03–6.97 (m, 4H), and 6.71 (d, *J* = 8.4 Hz, 4H).

4-(((1*S*,4*S*)-5,6-Bis(4-hydroxyphenyl)-*N*-(2,2,2-trifluoroethyl)-7-oxabicyclo [2.2.1]hept-5-ene)-2-sulfonamido)phenyl 3-(2-(2-(2-(2-((*E*)-2-(4-(dicyanomethyl-ene)-4*H*-chromen-2-yl)vinyl)-5-(diethylamino)phenoxy)ethoxy)ethoxy)ethoxy)-propanoate (**OBHSA-PEG-DCM**):

Compounds **9** (135 mg, 158.65 mmol) and **10** (40 mg, 158.65 mmol) were dissolved in anhydrous tetrahydrofuran (100 mL) under a nitrogen atmosphere. The reaction mixture was then refluxed for 12 h. Then, the resulting mixture was extracted with EA and H_2_O. The organic phase was concentrated and further purified by column chromatography (CH_2_Cl_2_/MeOH = 150:1) to yield **OBHSA-PEG-DCM** (117.3 mg, yield: 67%). ^1^H NMR (400 MHz, DMSO-*d*_6_) δ 9.71 (s, 1H), 9.68 (s, 1H), 8.72–8.65 (m, 1H), 7.90–7.81 (m, 2H), 7.70–7.63 (m, 1H), 7.57–7.50 (m, 2H), 7.46–7.36 (m, 2H), 7.14–7.04 (m, 7H), 6.76–6.70 (m, 3H), 6.70–6.66 (m, 2H), 6.41–6.31 (m, 1H), 6.28 (d, *J* = 2.3 Hz, 1H), 5.47 (d, *J* = 1.2 Hz, 1H), 5.33–5.29 (m, 1H), 4.57 (d, *J* = 9.1 Hz, 2H), 4.26–4.15 (t, *J* = 4.4 Hz, 2H), 3.89–3.83 (t, *J* = 4.5 Hz, 2H), 3.74–3.68 (m, 2H), 3.67–3.63 (t, *J* = 6.2 Hz, 2H), 3.60 (s, 3H), 3.55–3.43 (m, 8H), 2.77–2.70 (t, *J* = 6.2 Hz, 2H), 1.87 (d, *J* = 6.7 Hz, 2H), and 1.14–1.09 (t, *J* = 7.0 Hz, 6H). ^13^C NMR (151 MHz, Chloroform-*d*) δ 169.9, 162.9, 160.6, 160.4, 157.3, 157.1, 156.5, 155.9, 153.2, 152.7, 151.4, 150.6, 141.1, 137.1, 136.7, 136.4, 134.2, 130.4, 129.7, 129.3, 128.4, 125.8, 125.7, 124.0 (q, *J* = 150.5 Hz), 123.0, 118.6, 118.3, 118.2, 116.3, 115.9, 115.7, 113.3, 112.1, 105.3, 105.1, 95.0, 84.8, 82.9, 80.0, 71.2, 70.8, 70.6, 70.5, 69.9, 67.8, 66.6, 61.8, 58.3, 52.9 (q, *J* = 75.5 Hz), 49.5, 44.9, 38.9, 36.8, 35.3, 34.1, 31.8, 25.7, 25.1, and 12.9. ^19^F NMR (376 MHz, DMSO-*d*_6_) δ −69.4. HRMS (ESI, positive) *m*/*z* calculated for [M + H]^+^ C_59_H_57_F_3_N_4_O_12_S: 1103.3646, found: 1103.3719.

### 4.2. HPLC Analysis

The liquid chromatography was performed using an Agilent 1260 HPLC system (Agilent Technologies, Santa Clara, CA, USA) equipped with a binary pump and a UV-Vis detector. Separation was achieved on a XAqua C18 column (250 × 4.6 mm, 5 μm) (XAqua, Guangzhou, China). The mobile phase consisted of solvent A (water with 0.1% TFA) and solvent B (acetonitrile). Gradient elution commenced with 20% A and 80% B, followed by a linear gradient to 90% A over 5 min, which was then held for 10 min. The flow rate was maintained at 1 mL/min, and the column temperature was set at 25 °C. Detection was performed at 254 nm.

### 4.3. Photophysical Properties

To assess the optical characteristics of OBHSA-PEG-DCM, UV and fluorescence spectra were obtained within a pH 7.4 PBS solution containing 10% DMSO, utilizing the SHIMADZU UV-2600 UV-vis spectrophotometer (Shimadzu Corporation, Kyoto, Japan) and the HITACHI F-4600 fluorescence spectrophotometer (Hitachi High-Tech Corporation, Tokyo, Japan). The DMSO stock solution of OBHSA-PEG-DCM (10 mM) was diluted into a solvent of DMSO/phosphate-buffered saline (PBS) (*v*/*v* = 10/90, pH 7.4) to give a final concentration of 1.0 μM. In the anti-interference experiment, the fluorescence intensity was measured at 682 nm with an excitation wavelength of 550 nm, using a HITACHI F-4600 (Slit = 10/10 nm, Hitachi High-Tech Corporation, Tokyo, Japan) at 37 °C.

### 4.4. Confocal Laser Scanning Microscopy

MCF-7 cells were cultured in MEM supplemented with 10% calf serum, penicillin (100 U/mL), and streptomycin (100 μg/mL) at 37 °C under a humidified 5% CO_2_-containing atmosphere. Subsequently, cells with a density of 4 × 10^5^ were loaded onto confocal Petri dishes with coverslips of a 35 mm diameter and cultured overnight. The cells were then incubated with OBHSA-PEG-DCM (1.0 μM) for 30 min, followed by the addition of DAPI for nucleus staining for 10 min. Fluorescence images of the stained cancer cells were captured using a confocal scanning microscope (CLSM, Nikon-A1 system, Nikon Corporation, Tokyo, Japan). OBHSA-PEG-DCM was visualized using a 550 nm laser, and the emission wavelength was detected within the range of 650 to 750 nm, represented in red fluorescence.

### 4.5. In Vitro Cell Culture and Antiproliferative Assay

Human breast cancer MCF-7 cells were obtained from ATCC. Cells were cultured in MEM containing 10% FBS and 1% penicillin–streptomycin. The antiproliferative activity of OBHSA-PEG-DCM on MCF-7 cell viability was assessed using the CCK-8 (Cell Counting Kit 8) assay (Beyotime, Shanghai, China). MCF-7 cells were seeded onto 96-well plates at a density of 1 × 10^4^ cells per well for stationary culture. Subsequently, varying concentrations of OBHSA-PEG-DCM were added to the cell culture medium. After 24 h of incubation, the cell culture medium was replaced with 100 μL of fresh culture medium, and 10 μL of CCK8 was added to each well. The optical density (OD) value was measured using a Biotek Synergy H2 microplate reader (BioTek Instruments, Winooski, VT, USA) at a wavelength of 450 nm. The IC_50_ was calculated using GraphPad Prism 9 software. The IC_50_, representing the concentration of compounds inducing a 50% growth inhibition, was determined. Each drug concentration was tested in four technical replicates and three biological replicates.

### 4.6. Western Blotting

To evaluate the ability of OBHSA-PEG-DCM to induce ERα degradation in MCF-7 cells using Western blotting (WB), MCF-7 cells were seeded in 6-well plates (1 × 10^6^ cells/well) and cultured at 37 °C for 24 h. Then, cells were treated with different concentrations of OBHSA-PEG-DCM for 48 h. Blank control and Fulvestrant control groups were also included. Cells were collected and washed with PBS. Cell lysis was performed using SDS lysis buffer (Beyotime, Shanghai, China), and the total protein was extracted. The protein samples were mixed with SDS-PAGE loading buffer and boiled for 5 min. Equal amounts of protein samples were loaded onto 8% SDS-PAGE gels for electrophoresis. After electrophoresis, the gels were transferred onto PVDF membranes. The PVDF membranes were incubated with ERα primary antibody (1:1000, CST) and GAPDH (1:8000, Proteintech Group) at 4 °C overnight. After washing with TBST, the membranes were incubated with HRP-labeled secondary antibody (Kerui Biotechnology Co., Ltd., Wuhan, China) at room temperature for 1 h. Finally, the blots were developed using an ECL chemiluminescent kit.

### 4.7. In Vivo Fluorescence Imaging

All animal operations in this article are in compliance with the relevant regulations and ethical requirements. Mice were maintained in standard housing conditions (10 h light/14 h dark cycle, 21~25 °C, 40~70% humidity). To establish a breast cancer model, we selected healthy female BALB/c mice (6~8 weeks old, SPF grade) and subcutaneously injected 2 × 10^6^ MCF-7 breast cancer cells into the right hind limb. The animals were cared for following the guidelines provided by the Wuhan University Experimental Animal Management Center. One week after subcutaneous tumor inoculation, when the tumor diameter reached 4~6 mm, mice were intravenously administered with OBHSA-PEG-DCM (dose: 5 nmol) dissolved in PBS containing 0.3% DMSO. Anesthesia was induced using 5% isoflurane, and the mice were placed in an ex vivo bioluminescence imaging system (PerkinElmer, Waltham, MA, USA) IVIS Lumina III. Fluorescence images were acquired at different time points following OBHSA-PEG-DCM injection, using an excitation wavelength of 480 nm and a filter at 580 nm. Quantitative analysis of tumor fluorescence images was performed using IVIS software (version 4.5).

### 4.8. H&E Staining

Balb/c mice were used in this study, with a total of 12 mice divided into two groups: a negative control group (4 mice) and an experimental group with equal numbers of males and females (4 males and 4 females). The experimental group received a drug treatment at a dosage of 100 mg/kg. The negative control group was treated with a solution of 5% DMSO, 45% PEG, and 50% PBS. After one week of treatment, the mice were sacrificed, and tissue samples from the heart, liver, lungs, and kidneys were collected for histological analysis.

After fixation of the mouse viscera in 4% paraformaldehyde for 24 h, dehydration and paraffin embedding were performed. Hematoxylin and eosin (H&E) staining was carried out on cell nuclei and cytoplasm according to the standard protocol. The viscera were observed using Image-Pro Plus software (Version 7.0) to determine the extent of tissue damage.

## Figures and Tables

**Table 1:** IC_50_ values of OBHSA and OBHSA-PEG-DCM in various human tumor cell lines *^a^*.

Compounds	MCF-7 (IC_50_, µM)	MDA-MB-231 (IC_50_, µM)	DU145 (IC_50_, µM)
OBHSA	0.99 ± 0.07	>100	>100
OBHSA-PEG-DCM	1.09 ± 0.08	6.60 ± 2.84	>100
DCM-PEG	>100	>100	>100

*^a^* Experimental values represent an average of at least three independent experiments ± standard deviation (mean ± SD).

## Data Availability

The data are available from the corresponding author upon reasonable request.

## Supplementary Materials

The following supporting information can be downloaded at: https://www.mdpi.com/article/10.3390/molecules30020305/s1. References [36,42] are cited in the Supplementary Materials.

## Abbreviations

BC, breast cancer; C, control; CCK-8, Cell Counting Kit-8; DCM, dicyanomethylene-4*H*-pyran; DDS, drug delivery system; EPR, enhanced permeability and retention; ER, estrogen receptor; 4-OHT, 4-Hydroxytamoxifen; ful, fulvestrant; GAPDH, Glyceraldehyde 3-phosphate dehydrogenase; OBHSA, 7-oxabicyclo [2.2.1]heptene sulfonamide; PEG, polyethylene glycol; PROTACs, Proteolysis-Targeting Chimeras; RBA, relative binding affinity; ROS, reactive oxygen species; SERDs, selective estrogen receptor degraders; SERMs, selective estrogen receptor modulators; TME, tumor microenvironment.
