# Supplementary material for: Pegylated NIR Fluorophore-Conjugated OBHSA Prodrug for ERα-Targeted Theranostics with Enhanced Imaging and Long-Term Retention
Source: Molecules. 2025 Jan 14;30(2):305. doi: 10.3390/molecules30020305 (PMC11767339; doi:10.3390/molecules30020305)
Supplement: Supplementary file 1 [file molecules-30-00305-s001.zip › molecules-3329576-supplementary.pdf]

Supporting Information

Pegylated NIR Fluorophore-Conjugated OBHSA Prodrug for ERα-Targeted Theranostics with Enhanced Imaging and Long-Term Retention

Xiaohua Wang [1,2], Xiaofei Deng [3], Lilan Xin [3], Chune Dong [3,4], Guoyuan Hu [1,*] and Hai-Bing Zhou [3,4,*]

1 School of Environmental Ecology and Biological Engineering, Wuhan Institute of Technology,
Wuhan 430205, China; msnhm123@gmail.com

2 College of Life Sciences, Wuchang University of Technology, Wuhan 430223, China

3 Department of Hematology, Zhongnan Hospital of Wuhan University, Wuhan University School of Pharmaceutical Sciences, Wuhan 430071, China

4 State Key Laboratory of Virology and Biosafety, Frontier Science Center for Immunology and Metabolism, Wuhan University School of Pharmaceutical Sciences, Wuhan 430071, China; dxfvca@whu.edu.cn (X.D.); 18537688335@163.com (L.X.); cdong@whu.edu.cn (C.D.)

* Correspondence: hgy701@163.com (G.H.); zhouhb@whu.edu.cn (H.-B.Z.); Tel.: +86-2768759586 (H.-B.Z.)

Table of Contents

Synthesis and characterization 3

HRMS Analysis of the Mechanistic Hydrolysis of OBHSA-PEG-OBHSA 5

Representative Spectra of NMR and HRMS 6

NOESY-NMR of OBHSA-PEG-DCM 13

Raw images of Western blot data 14

Relative Affinity of DCM for ERα and ERβ 15

References 16

Synthesis and characterization

Key intermediate, (*E*)-2-(2-(4-(diethylamino)-2-hydroxystyryl)-4*H*-chromen-4-ylidene)malononitrile **5**, was synthesized using established protocols with necessary modifications[1, 2].

1-(2-Hydroxyphenyl)butane-1,3-dione (2).

To a solution of 1-(2-hydroxyphenyl)ethan-1-one (**1**) (5.00 g, 36.7 mmol) dissolved in 100 mL of anhydrous THF, 8 mL of EtOAc was added. NaH (3.53 g, 146.9 mmol) was slowly introduced under N_2 conditions at 0 °C. The reaction mixture was stirred at room temperature for 30 min and then heated to reflux for 2 h, resulting in the formation of a greyish solid product. The mixture was poured into 100 mL of ice water, and the pH of the solution was adjusted to neutral. The solution was extracted with EtOAc, and the organic phase was dried over Na_2SO_4, filtered, and concentrated. This process yielded compound **2** as a brown sticky solid, which was used in the subsequent reaction without further purification.

2-Methyl-4*H*-chromen-4-one (3).

Compound **2** was (3 g, 16.8 mmol) was dissolved in a mixture of acetic acid (50 mL) and sulfuric acid (2 mL), and refluxed at 120 °C for about 45 min. Subsequently, the reaction mixture was poured into ice water, and the pH was adjusted to neutral using a saturated Na_2CO_3 solution. The aqueous solution was extracted with CH_2Cl_2, dried over anhydrous Na_2SO_4, filtered, and concentrated. The obtained crude product was purified by column chromatography (PE/Et_2O: 10/2) to yield compound **3** as an off-white solid (6.2 g, yield, 85%). ^{1}H NMR (400 MHz, DMSO-d_6) δ 8.03 – 7.96 (m, 1H), 7.81 – 7.72 (m, 1H), 7.59 (d, J = 8.4 Hz, 1H), 7.50 – 7.41 (t, J = 7.5 Hz, 1H), 6.24 (s, 1H), 2.38 (s, 3H).

2-(2-Methyl-4*H*-chromen-4-ylidene)malononitrile (4).

Malononitrile (12.0 g, 449.5 mmol) was introduced into a solution containing compound **3** (6 g, 37.5 mmol) and acetic anhydride (80 mL), followed by reflux at

140 °C for 14 h. Afterward, the reaction mixture was concentrated, and 100 mL of distilled water was added, with subsequent reflux for an additional 30 min. The resulting mixture was then subjected to extraction using CH_2Cl_2, dried over anhydrous Na_2SO_4, filtered, and concentrated. The resulting crude product underwent purification via column chromatography (PE/Et_2O: 8/2), yielding compound **4** as a reddish-orange solid (5.4 g, yield: 69%). ^1H NMR (400 MHz, Chloroform-*d*) δ 8.94 – 8.87 (m, 1H), 7.76 – 7.68 (m, 1H), 7.49 – 7.40 (m, 2H), 6.70 (s, 1H), 2.44 (s, 3H).

(*E*)-2-(2-(4-(diethylamino)-2-hydroxystyryl)-4*H*-chromen-4-ylidene)-malononitrile (5).

Under an argon atmosphere, compound 4 (5.0 g, 24.0 mmol) and 4-(diethylamino)-2-hydroxybenzaldehyde (5.1 g, 26.4 mmol) were introduced into a 100 mL toluene solution, followed by the addition of equimolar amounts of piperidine (0.4 mL) and acetic acid (0.4 mL). The reaction mixture was heated to 120 °C and refluxed for 12 h. Subsequently, the mixture was concentrated to an appropriate volume and extracted with CH_2Cl_2. The combined organic layer underwent washing with water and brine, drying over Na_2SO_4, filtration, and concentration. The resulting crude product was further purified through column chromatography (CH_2Cl_2/MeOH = 100:1) to yield compound **5** (7.7 g, yield: 84%). 1H NMR (400 MHz, DMSO-d_6) δ 10.13 (s, 1H), 8.70 (d, J = 8.3 Hz, 1H), 7.91 (d, J = 15.8 Hz, 1H), 7.88 – 7.80 (m, 1H), 7.75 (d, J = 8.4 Hz, 1H), 7.59 – 7.50 (m, 2H), 7.01 (d, J = 15.8 Hz, 1H), 6.77 (s, 1H), 6.33 – 6.26 (m, 1H), 6.17 (d, J = 2.4 Hz, 1H), 3.39 (s, 4H), 1.16 – 1.08 (t, J = 6.9 Hz, 6H).

HRMS Analysis of the Mechanistic Hydrolysis of OBHSA-PEG-OBHSA

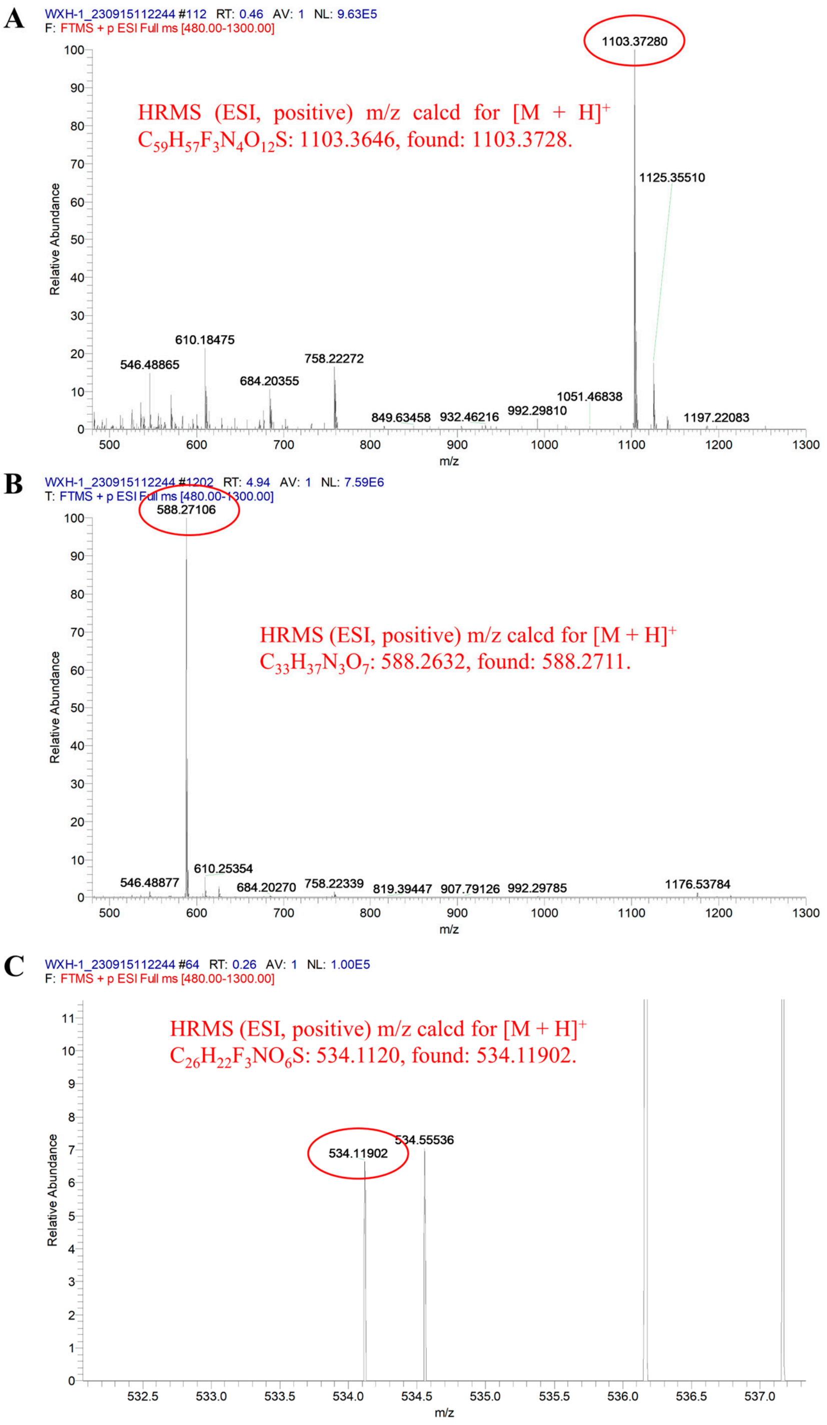

Figure S1. HRMS analysis of cell lysates after co-incubation of 10 µM **OBHSA-PEG-DCM** with MCF-7cells for 45 min. (A) **OBHSA-PEG-DCM**. (B) pegylated DCM. (C) OBHSA.

Representative Spectra of NMR and HRMS

^{1}H NMR spectrum of **3**

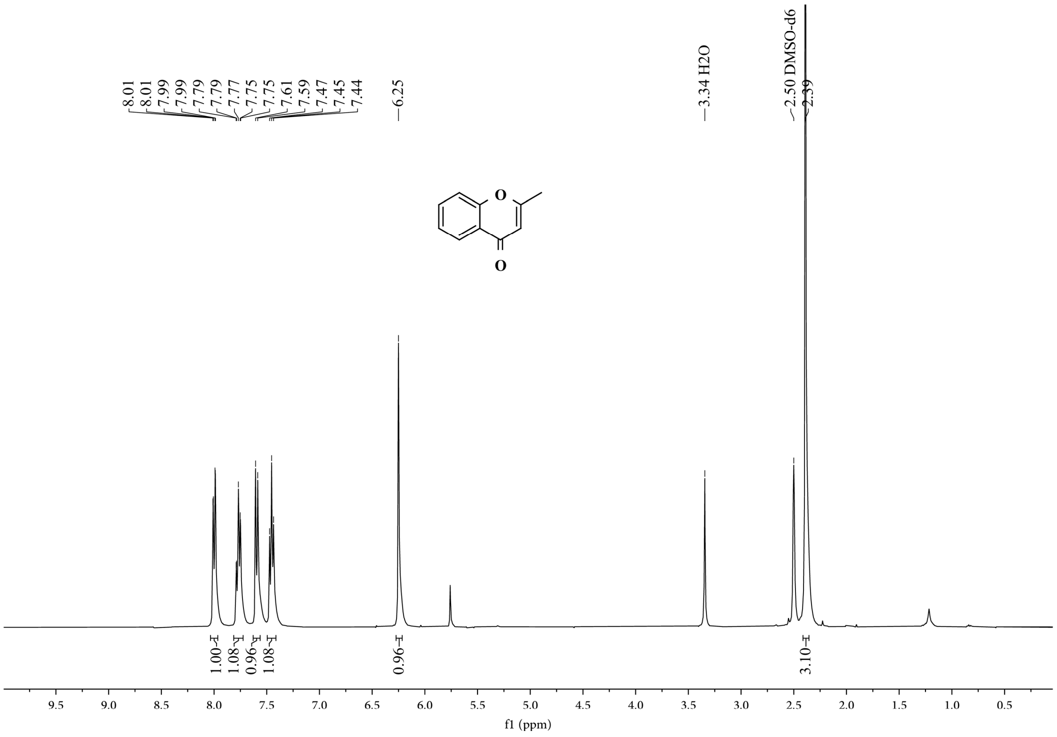

^{1}H NMR spectrum of **4**

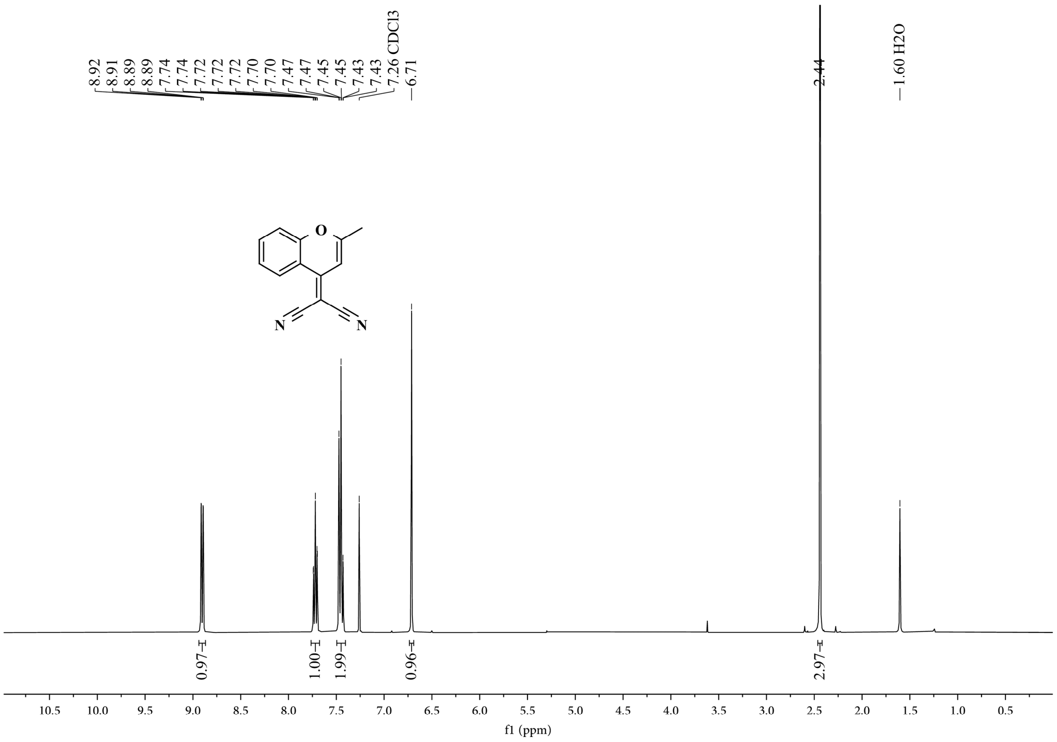

^{1}H NMR spectrum of **5**

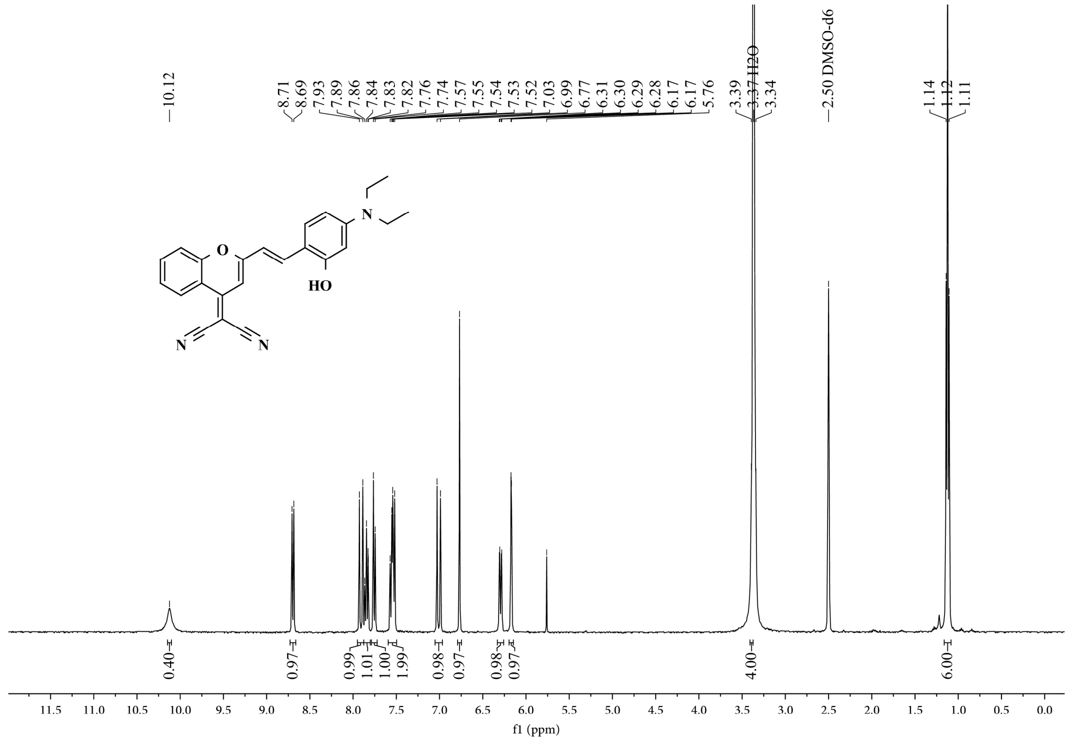

^{1}H NMR spectrum of **6**

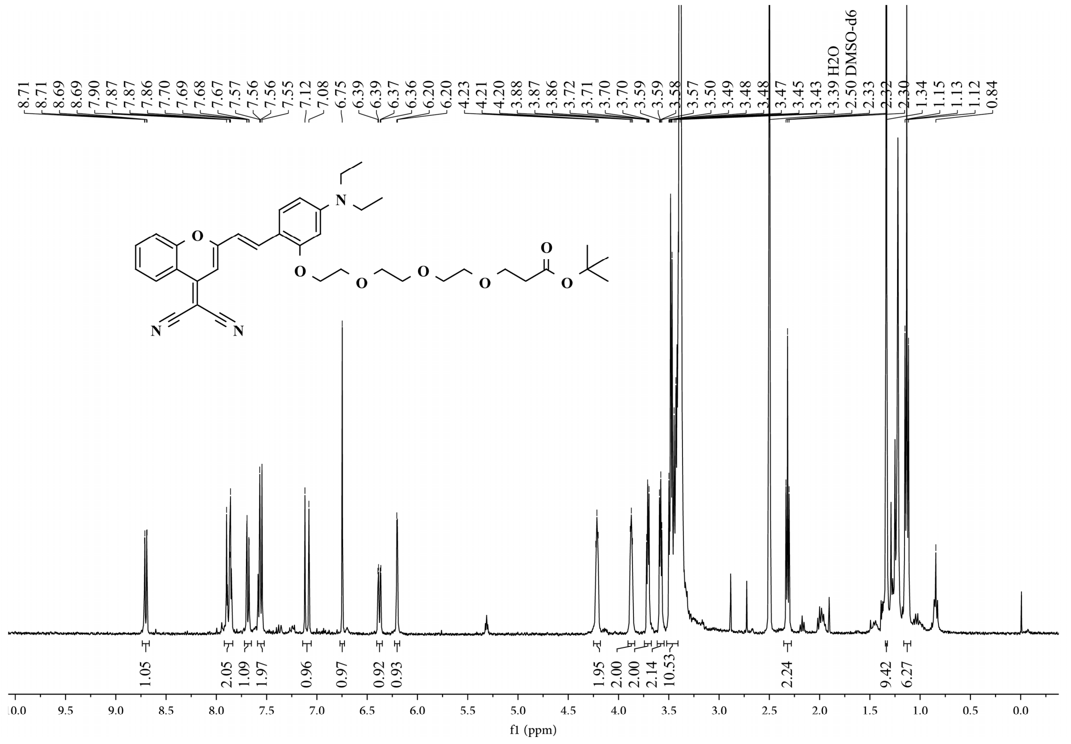

^{1}H NMR spectrum of **7**

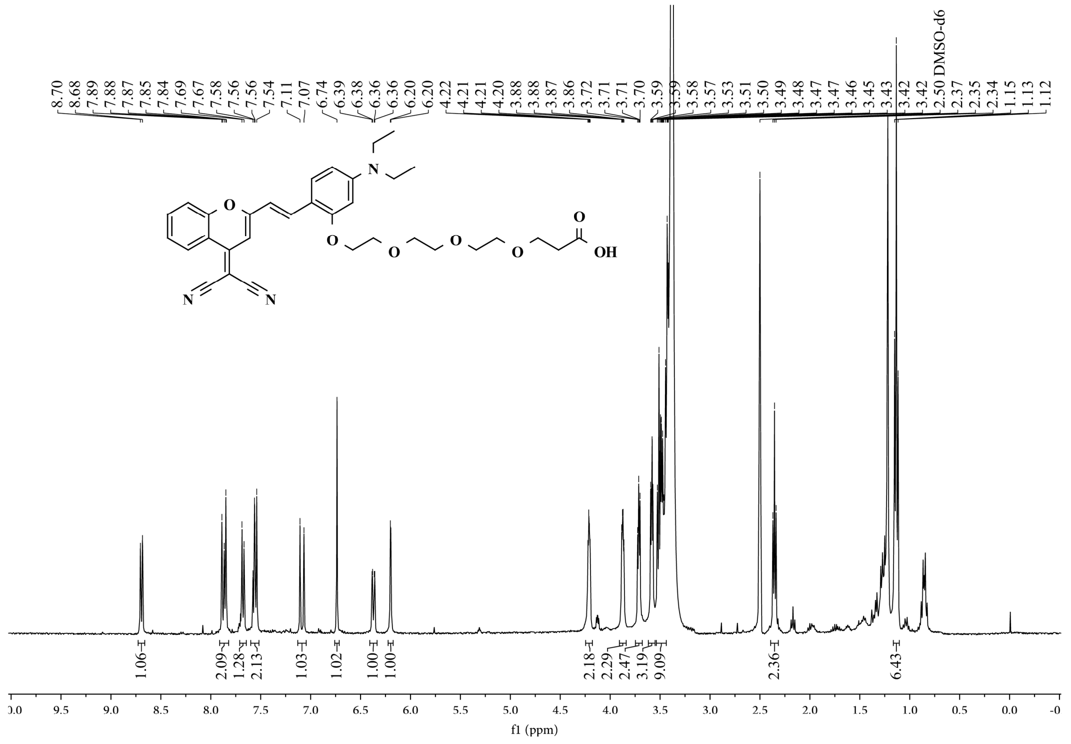

^{1}H NMR spectrum of **8**

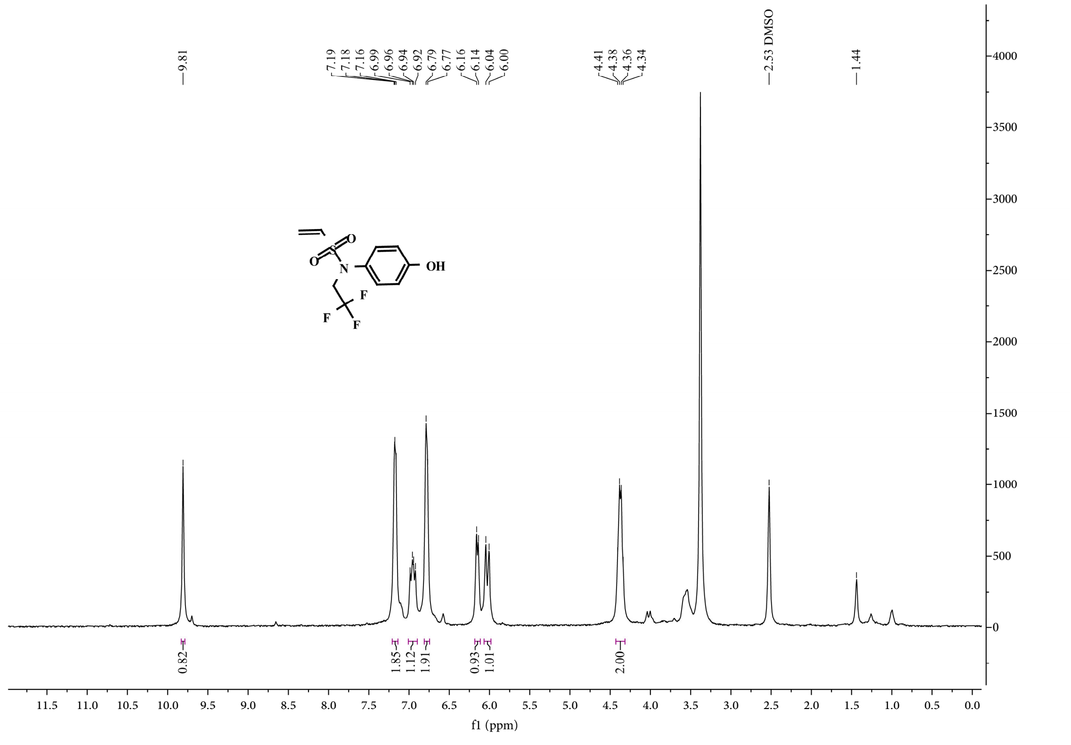

^{1}H NMR spectrum of **9**

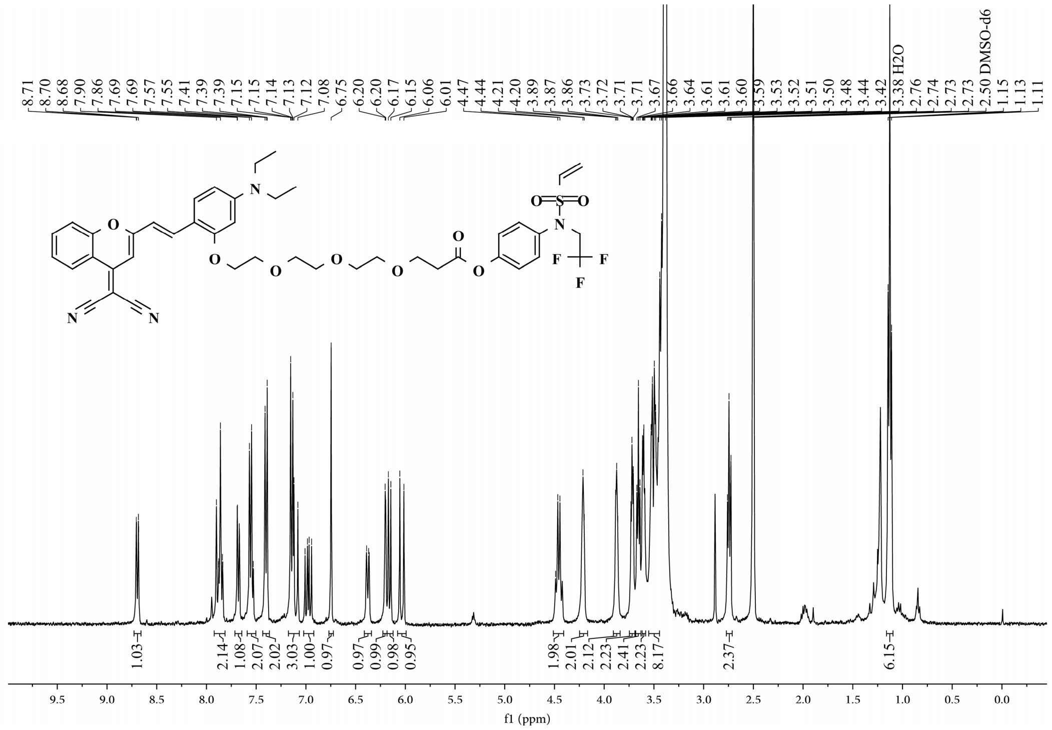

^{1}H NMR spectrum of **OBHSA-PEG-DCM**

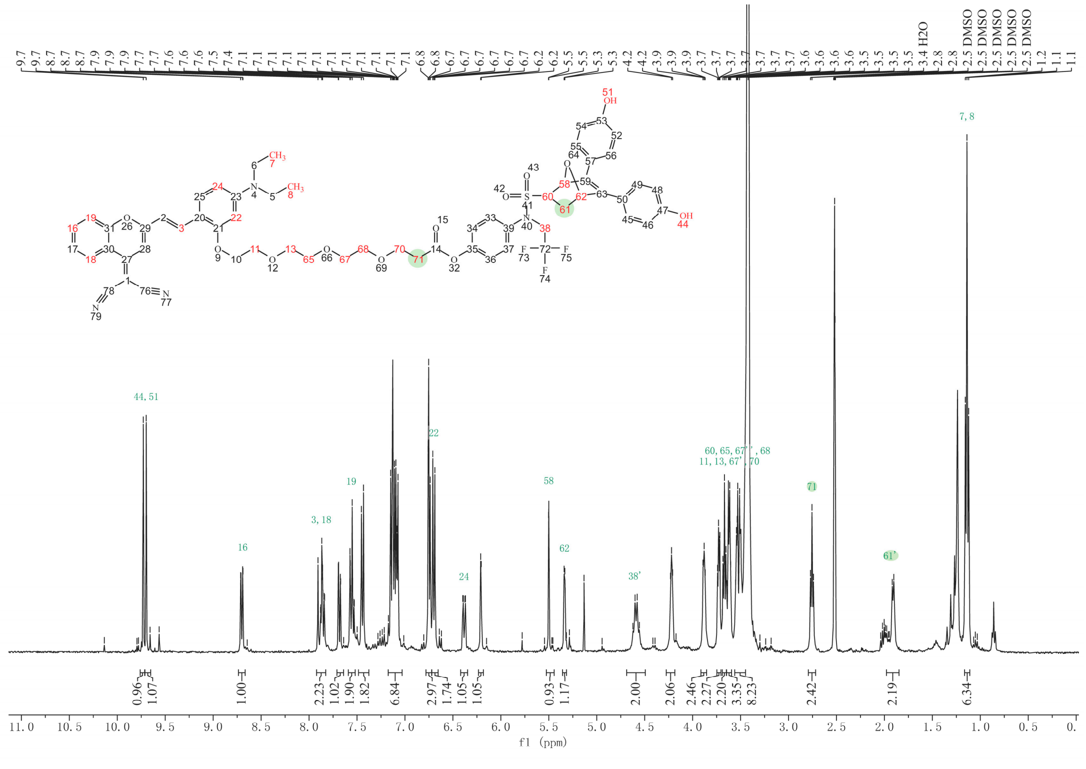

^{13}C NMR spectrum of **6**

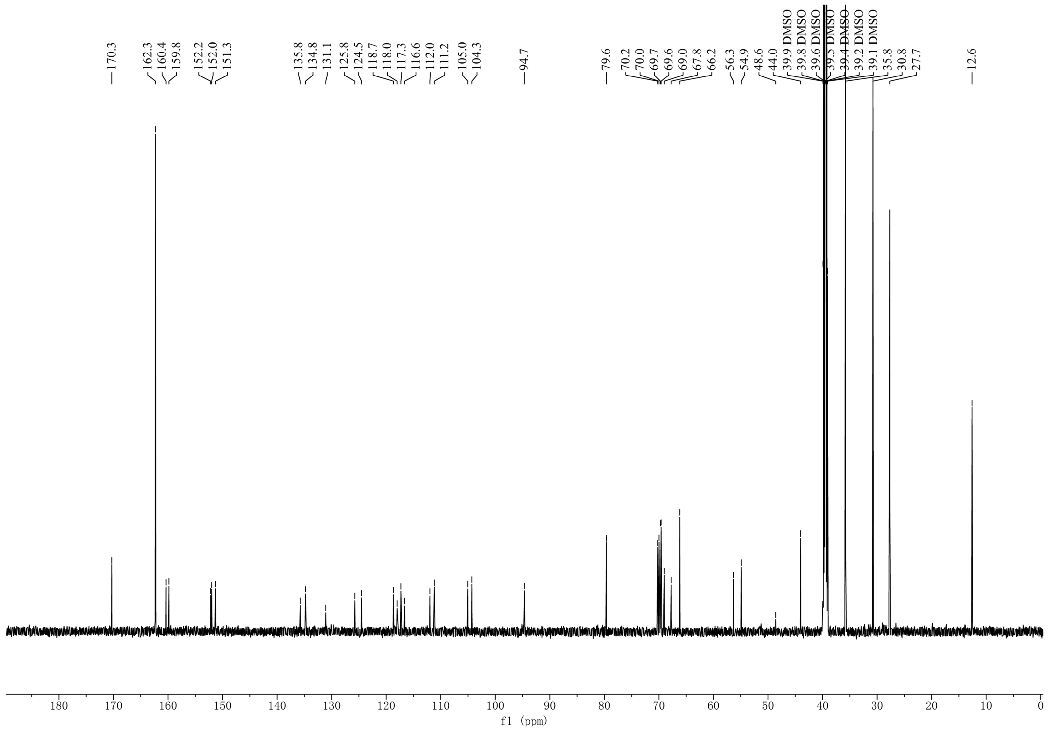

^{13}C NMR spectrum of **9**

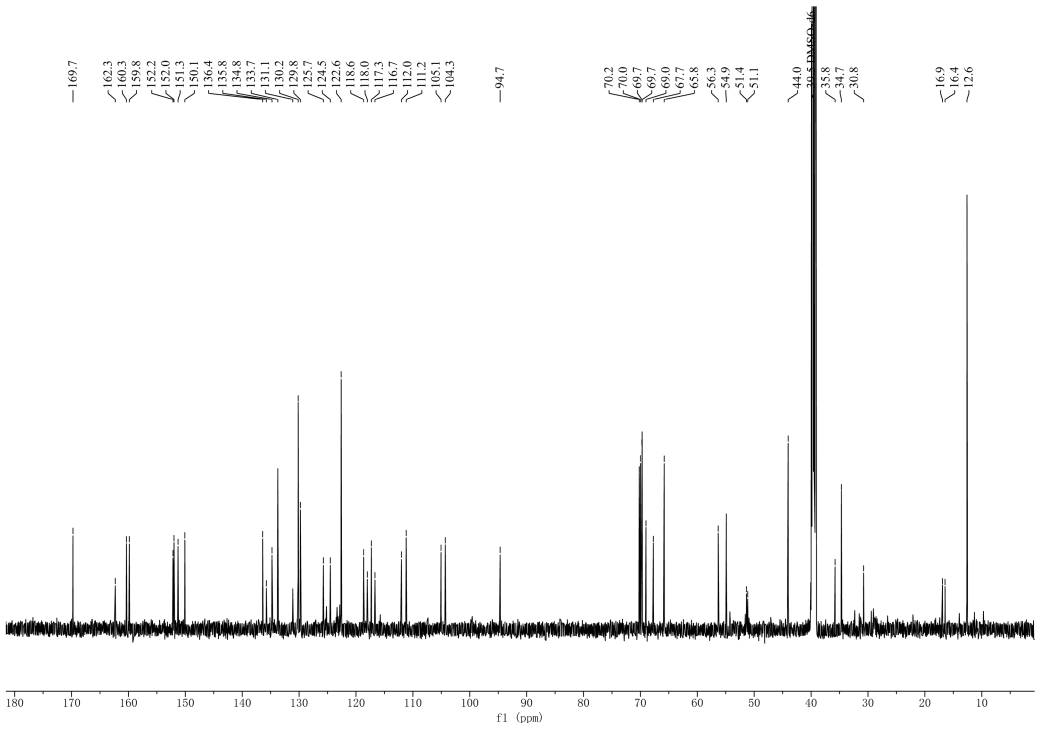

^{13}C NMR spectrum of **OBHSA-PEG-DCM**

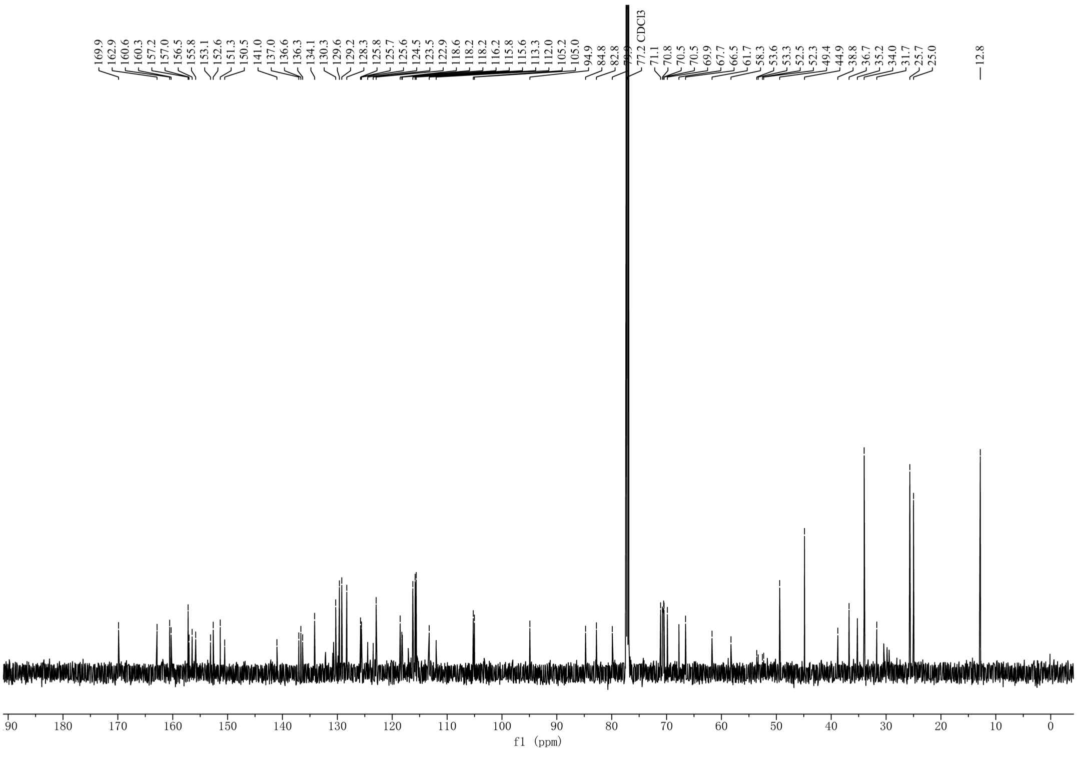

^{19}F NMR spectrum of **9**

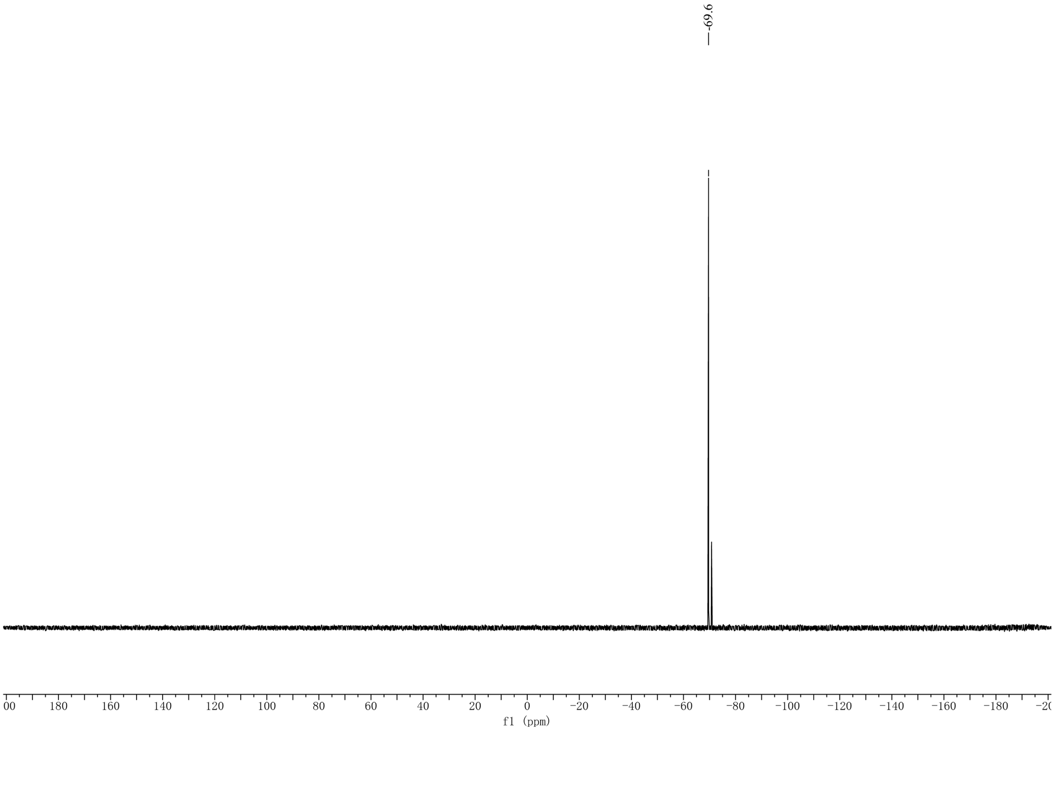

^{19}F NMR spectrum of **OBHSA-PEG-DCM**

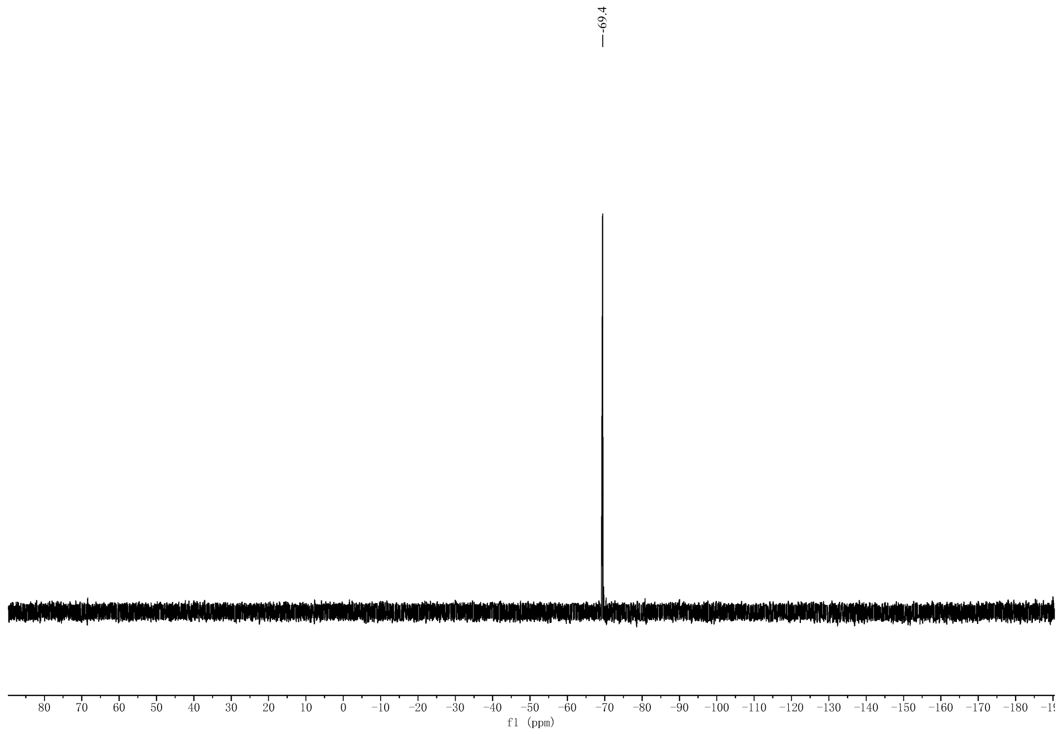

Mass spectrum of **OBHSA-PEG-DCM**

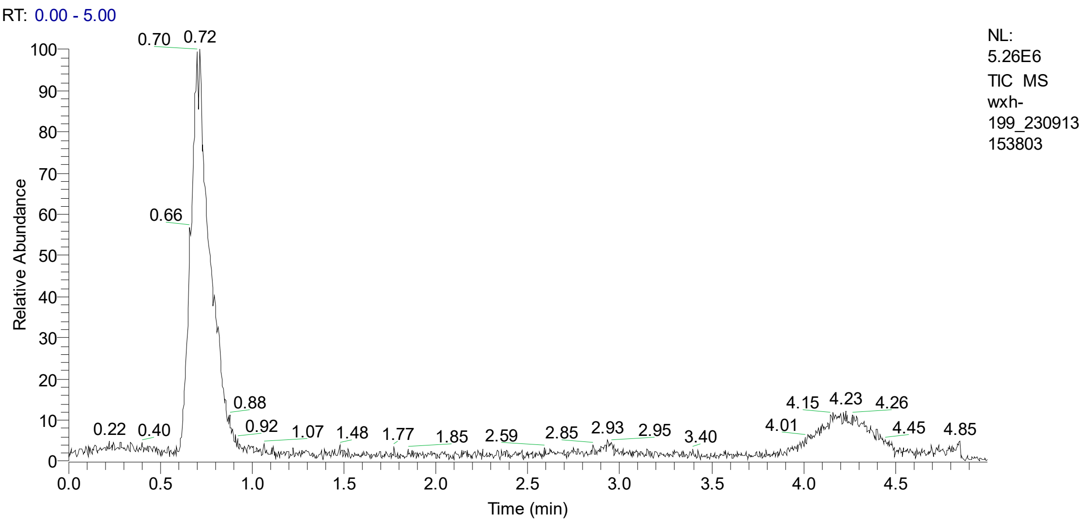

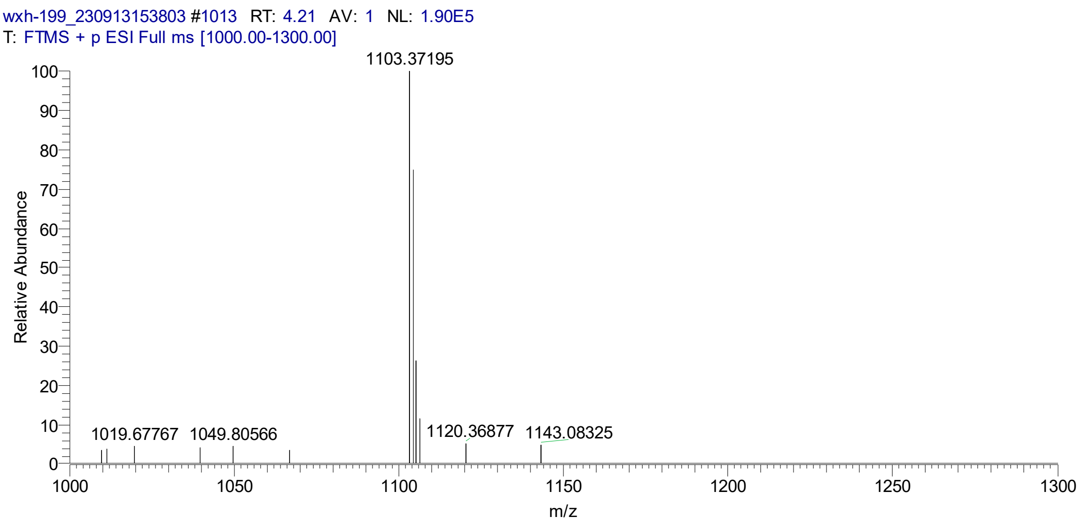

NOESY-NMR of OBHSA-PEG-DCM

The peaks at δ 5.32 (H_1) and δ 5.48 (H_2) correspond to the hydrogen atoms on the bridgehead carbon. It is evident that H_2 interacts with the hydrogen atoms of NCH_2CF_3 (δ 4.56). The hydrogen atoms of NCH_2CF_3 (δ 4.56) interact with the two hydrogen atoms on the benzene ring (δ 7.04, 7.05). The NOESY-NMR spectra of **OBHSA-PEG-DCM** are shown as Fig. S2.

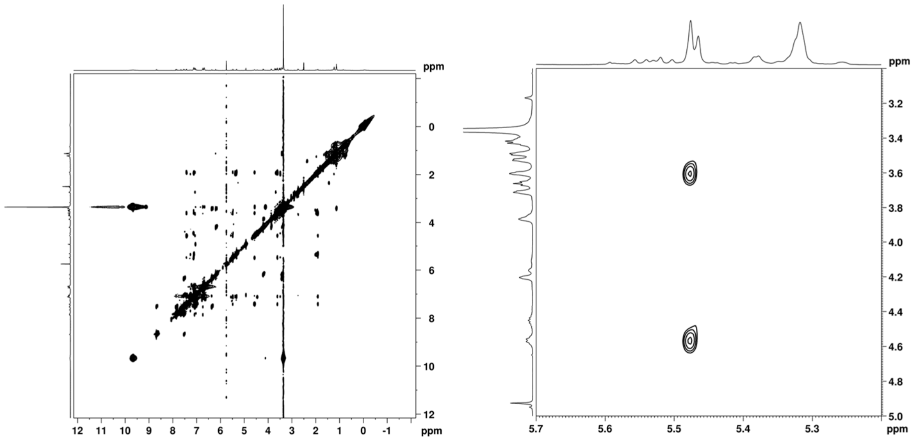

Figure S2 NOESY-NMR of OBHSA-PEG-DCM.

Raw images of Western blot data

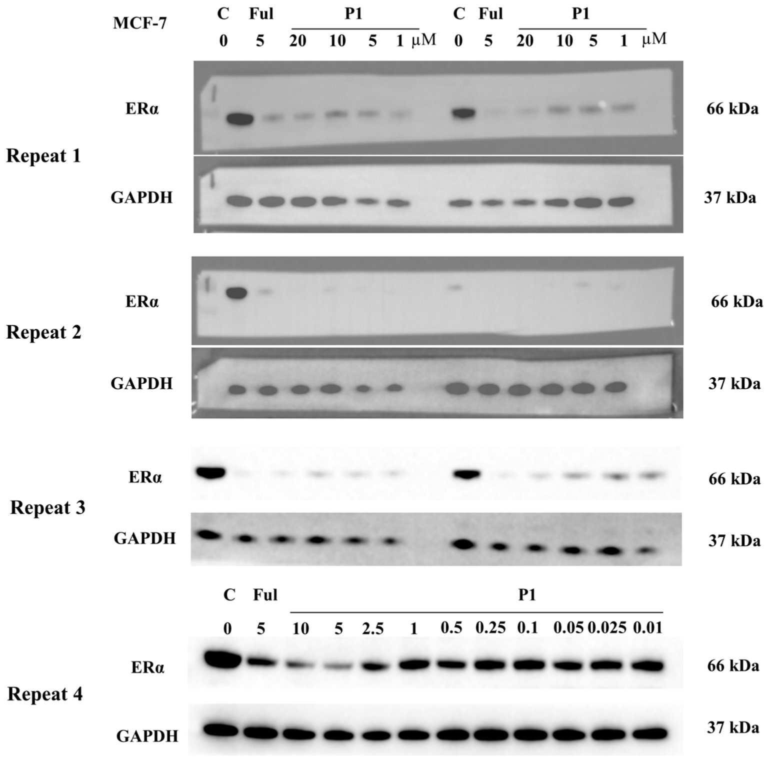

Figure S3. Original western blot evaluating the degradation activity in four repeats.

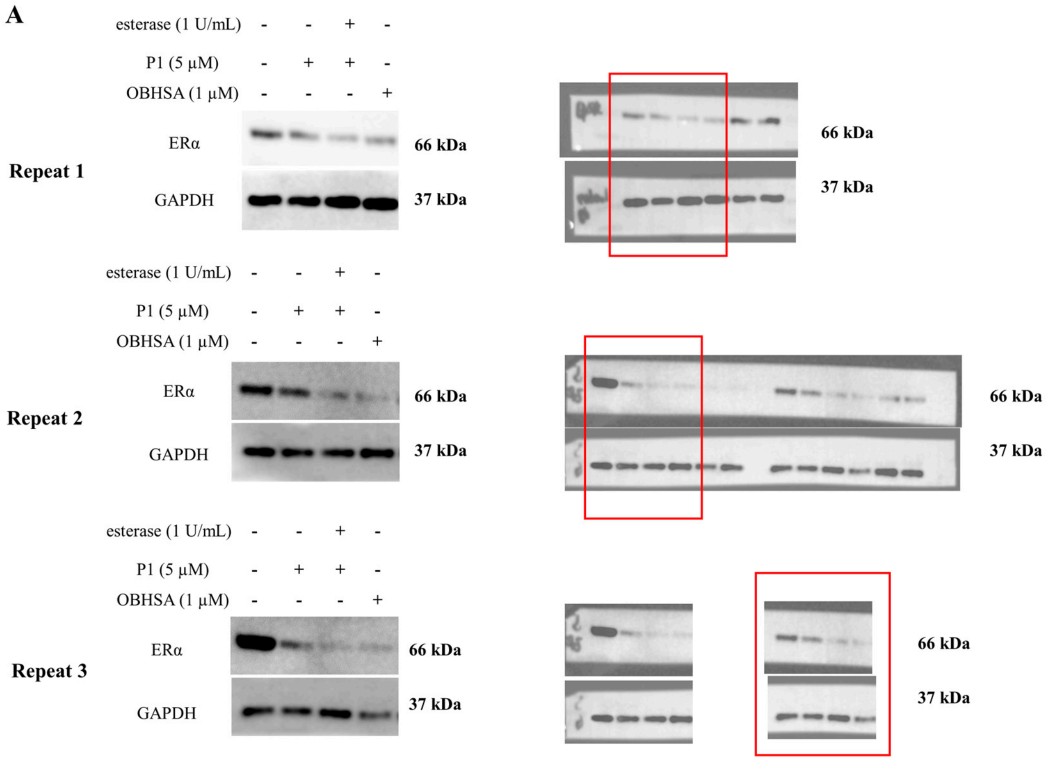

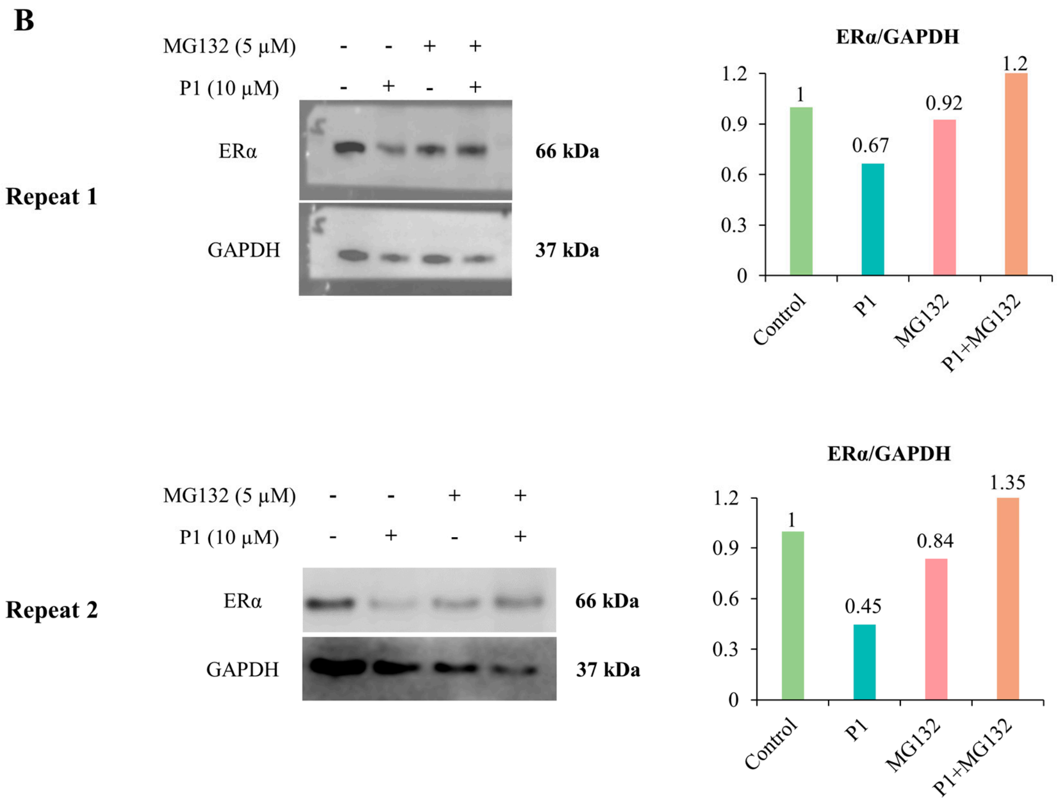

Figure S4. (A) Repeated experiments evaluation of the degradation activity of P1 and OBHSA. (B) Repeated experiments investigation of the degradation mechanism of P1. MCF-7 cells were treated with compound P1, MG132, or their combination for 24 hours, and the ERα level was then analyzed.

Relative Affinity of DCM for ERα and ERβ

Table S1 Relative Affinity of DCM for ERα and ERβ [a]

Structure	RBA (estradiol = 100)		
	**ERα**	**ERβ**	**β/α ratio**
	0.035 ± 0.021	0.011 ± 0.035	0.967

[a] Relative binding affinity (RBA) values are determined by competitive radiometric binding assays and are expressed as $IC_{50}^{estradiol}$ /$IC_{50}^{compound}$ × 100 ± the range or standard deviation (RBA, estradiol = 100%).

References

1. Zheng, Y. F.; Zhu, M. H.; Srinivasan, S.; Nwachukwu, J. C.; Cavett, V.; Min, J.; Carlson, K. E.; Wang, P. C.; Dong, C. N.; Katzenellenbogen, J. A.; Nettles, K. W.; Zhou, H. B., Development of Selective Estrogen Receptor Modulator (SERM)-Like Activity Through an Indirect Mechanism of Estrogen Receptor Antagonism: Defining the Binding Mode of 7-Oxabicyclo 2.2.1 hept-5-ene Scaffold Core Ligands. *Chemmedchem* **2012,** 7, (6), 1094-1100.
2. Rajasekhar, K.; Achar, C. J.; Govindaraju, T., A red-NIR emissive probe for the selective detection of albumin in urine samples and live cells. *Organic & Biomolecular Chemistry* **2017,** 15, (7), 1584-1588.
